# Genetic insights into therapeutic targets for gout: evidence from a multi-omics mendelian randomization study

**DOI:** 10.1186/s41065-024-00362-8

**Published:** 2024-12-30

**Authors:** Mingyuan Fan, Zhangjun Yun, Jiushu Yuan, Sai Zhang, Hongyan Xie, Dingyi Lu, Haipo Yuan, Hong Gao

**Affiliations:** 1 Hospital of Chengdu University of Traditional Chinese Medicine, Chengdu, Sichuan China; 2 Dongzhimen Hospital, Beijing University of Chinese Medicine (BUCM), Beijing, China; 3 TCM Regulating Metabolic Diseases Key Laboratory of Sichuan Province, Hospital of Chengdu University of Traditional Chinese Medicine, Chengdu, China; 4 Department of Endocrinology, Hospital of Chengdu University of Traditional Chinese Medicine, Chengdu, China

**Keywords:** Gout, Genetics, Drug target, MR analysis, Multi-omics

## Abstract

**Background:**

Considering that the treatment of gout is poor, we performed a Mendelian randomization (MR) study to identify candidate biomarkers and therapeutic targets for gout.

**Methods:**

A drug-targeted MR study was performed for gout by integrating the gout genome-wide association studies (GWAS) summary data and cis expression quantitative trait loci of 2,633 druggable genes from multiple cohorts. Summary data-based Mendelian randomization (SMR) analyses based on transcript and protein levels were further implemented to validate the reliability of the identified potential therapeutic targets for gout. Phenome-wide MR (Phe-MR) analysis was conducted in 1403 diseases to investigate incidental side effects of potential therapeutic targets for gout.

**Results:**

Eight potential therapeutic targets (ALDH3B1, FCGR2B, IL2RB, NRBP1, RCE1, SLC7A7, SUMF1, THBS3) for gout were identified in the discovery cohort using MR analysis. Replication analysis and meta-analysis implemented in the replication cohort validated the robustness of the MR findings (*P* < 0.05). Evidence from the SMR analysis (*P* < 0.05) further strengthened the reliability of the 8 potential therapeutic targets for gout also revealed that high levels of ALDH3B1 reduced the gout risk possibly modified by the methylation site cg25402137. SMR analysis (*P* < 0.05) at the protein level added emphasis on the impact of the risk genes NRBP1 and SUMF1 on gout. Phe-MR analysis indicated significant causality between 7 gout causal genes and 45 diseases.

**Conclusion:**

This study identified several biomarkers associated with gout risk, providing new insights into the etiology of gout and promising targets for the development of therapeutic agents.

**Supplementary Information:**

The online version contains supplementary material available at 10.1186/s41065-024-00362-8.

## Key Points

Identification of 8 potential therapeutic targets for gout, providing new avenues for drug development.

Integrated genomics, and proteomics to gain a comprehensive understanding of gout pathogenesis.

The identified targets and pathways offer new perspectives for clinical intervention and drug design.

**Supplementary Information:**

The online version contains supplementary material available at 10.1186/s41065-024-00362-8.

## Introduction

As the most common cause of inflammatory joint disease worldwide [1], gout is caused by high serum uric acid (UA) levels leading to deposition of monosodium urate crystals in the joints and other tissues, forming urate crystals and triggering inflammatory arthritis. Gout increases the risk of premature death from cardiovascular disease, chronic kidney disease, and diabetes [2]. Despite the existence of certain treatments, the prevalence and incidence of gout has continued to increase over the past few decades, with prevalence rates ranging from < 1–6.8%, and incidence rates ranging from 0.58 ~ 2.89/1000 person-years and increasing with age [2].

Current therapeutic strategies for gout focus on xanthine oxidase inhibitors (allopurinol or febuxostat), which inhibit urate production, and pro-uric acid excretion (probenecid or benzbromarone). Despite the effectiveness of current treatments against gout, a clinical study showed that only 40% of patients treated with allopurinol 300 mg/day achieved serum UA levels < 6.0 mg/dL [3]. With the use of febuxostat 80–240 mg/day, nearly one-third of patients entering clinical trials still did not achieve or maintain serum level goals [4]. This means that pharmacologic management of gout is often hard to succeed in getting patients to meet serum urate goals, with major dosing limitations and adverse effects. As research continues to identify the comorbidities associated with gout [2], current pharmacologic intervention protocols do not provide better control of acute gouty attacks in the presence of comorbidities [5]. The development of drugs targeting new mechanisms of gout may provide additional research directions for the current bottlenecks in intervention.

It is commonly believed that gout is associated with a poor lifestyle, but with deeper research into the pathomechanisms, the latest scientific evidence reveals that lifestyle has little impact on gout and is more influenced by increasingly well-defined genetic factors [6]. Previous studies have shown that genetic evidence based on human genetics identifies new pharmacologic targets and supports two-thirds of the drugs approved by the Food and Drug Administration (FDA) in 2021 [7]. Thus, drug development against genetic targets for gout may be a potential strategy for future treatment. Recently, Mendelian randomization (MR) studies have been widely used in studies of causal relationships between genetic exposures to disease and outcomes. Through the use of genome-wide association studies (GWAS), disease-associated SNPs can be mapped to genes encoding druggable targets, and genetic causality associated with gout can be unambiguously revealed at the gene and protein levels, helping to identify and validate potential drug targets for gout, addressing one of the key steps in new drug development that limits productivity and creativity. In this study, we aimed to identify potential therapeutic targets for gout by systematically investigating the causal association of druggable genes with gout risk through two-stage MR analysis.

## Methods and materials

### Study design

**Fig. 1:**
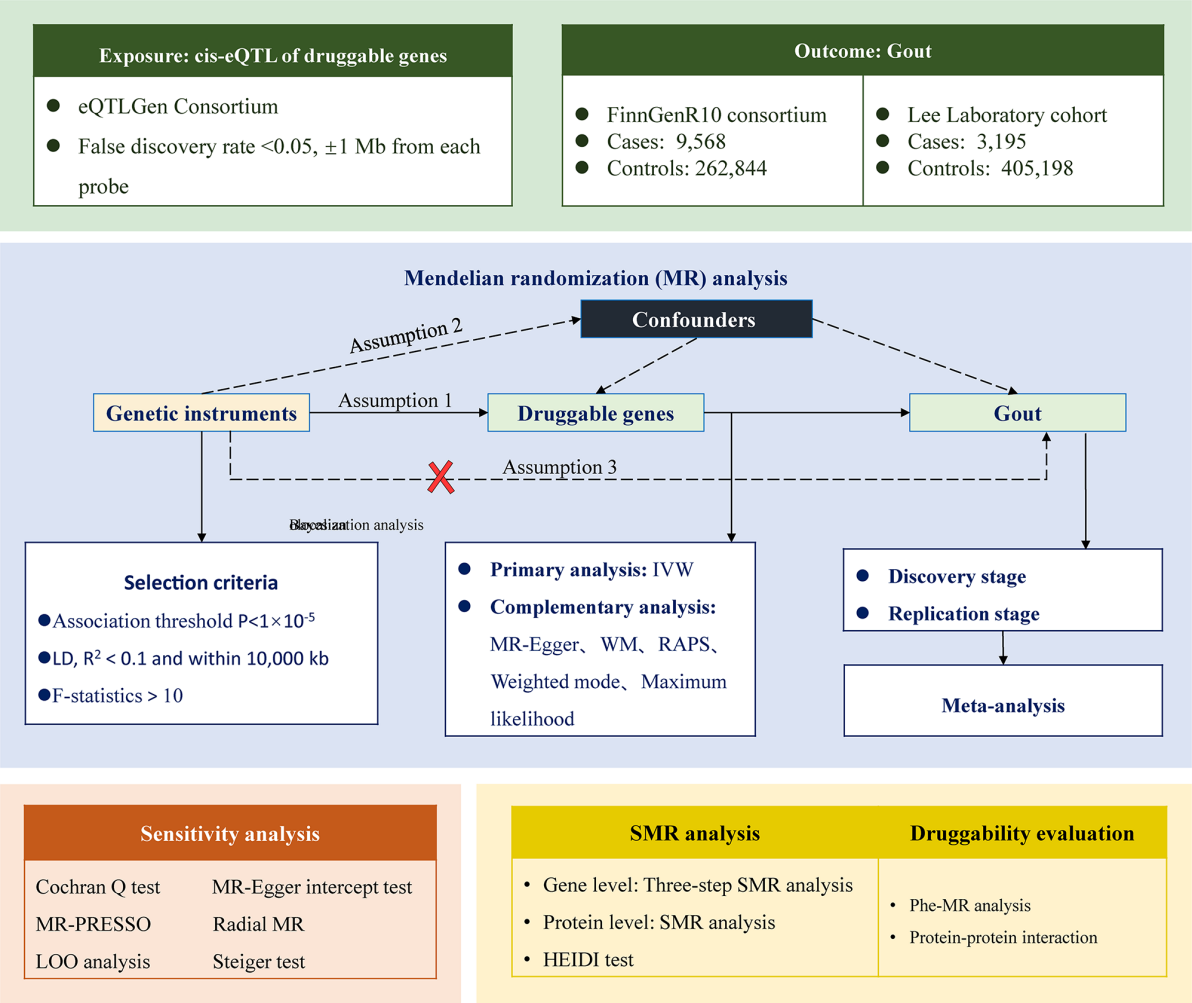
Schematic diagram of the datasets and analyses

Figure 1 shows the overall design of this study. The current MR analyses were all based on publicly available datasets. ln this study, we first extracted the genetic instrumental variables of the druggable genes and analyzed them by performing MR analyses with two independent gout datasets to initially identify drug candidate genes. A series of sensitivity analyses were then performed. Next, a three-step Summary data-based Mendelian randomization (SMR) analysis was used to determine the association between gene methylation and gout. Finally, for the identified drug targets, phenome-wide MR (Phe-MR) and protein-protein interaction (PPI) network were performed to assess their druggability.

### Data sources

#### Identifying cis-eQTL data linked to druggable genes

In reference to Finan’s study [8], a total of 4302 druggable genes with HGNC names were found. During the drug development phase, considering that cis-eQTL are closer to the gene of interest, we obtained cis-eQTL with full statistical significance from the eQTLGen Consortium (eQTL meta-analysis of peripheral blood from 31,684 individuals) (false discovery rate < 0.05, ± 1 Mb from each probe) [9]. For genetic tools representing 4302 druggable genes, we selected cis-eQTL within ± 100 kb from the genomic region of each gene. We calculated the proportion of explained variance (PVE) and F-statistic for each SNPs and excluded weak genetic variants with F-statistic<10, calculated as follows [10]:

$$\:PVE=\frac{{beta}^{2}}{{beta}^{2}+N*{se}^{2}}$$

$$\:F=\frac{N-k-1}{k}\times\:\frac{PVE}{1-PVE}$$

After clustering by linkage disequilibrium (R^2^ < 0.1 in a 10,000 kb window), 32,481 genetic variants for 2,645 druggable genes were finally obtained (Table S1).

#### GWAS data for gout

Gout GWAS summary data were provided by the FinnGenR10 consortium [11]. The Gout GWAS summary data used in this study contained 9,568 cases and 262,844 controls. To further validate the robustness of the findings and to expand the study sample size, we conducted a replication analysis using gout GWAS summary data (3,195 cases and 405,198 controls) from Lee Lab cohort (https://www.leelabsg.org/resources). Notably, there was no overlap in the gout GWAS samples from these two cohorts.

### Statistical analyses

First, we performed data harmonization to reconcile the effects of SNPs on exposure and outcome to ensure that β-values signed for the same allele. Next palindromic SNPs with intermediate allele frequencies (> 0.42) were eliminated. In addition, we used Steiger filtering to remove genetic variants that explained more of the outcome than the exposure. When IVs contained only 1 SNP, the Wald ratio method was selected for the primary MR analysis. For IVs containing 2 or more SNPs, the inverse variance weighting (IVW) method based on a multiplicative random effects model was selected for the primary analysis. IVW summarizes the Wald ratios analyzed across all genetic variants and provides unbiased estimates based on the assumption that there is no horizontal multiplicity across all SNPs [12].

To obtain more reliable MR results, we applied five MR methods based on different assumptions as a complementary analysis, including MR Egger [13], weighted median [14], robust adjusted profile score (RAPS) [15], weighted mode [16], maximum likelihood [17]. The direction of the estimates of the five MR models is consistent with the direction of the estimates of the IVW model is considered as the MR results are robust. Furthermore, a series of sensitivity analyses were implemented in this study to ensure the robustness of MR estimates, including Cochran’s Q test, MR-Egger intercept test, MR-Pleiotropy RESidual Sum and Outlier (MR-PRESSO), Radial MR and leave-one out (LOO) analysis. All MR analyses in this work were implemented based on TwoSampleMR 0.5.8 and RadialMR 1.1 of R 4.3.3.

Throughout the study, we used Bonferroni adjustments, Benjamini-Hochberg (BH) adjustment and q-value estimation based on the IVW multiple comparison method as the primary MR analysis to initially identify potential therapeutic targets for gout in the FinnGen cohort. Five additional MR models and a series of sensitivity analyses were used to validate the robustness of the MR results. Next, for the identified candidate targets, replication analyses were performed with a gout GWAS summary data from the Lee Lab cohort. Finally, a meta-analysis of IVW model estimates from both cohorts was performed to identify potential therapeutic targets for gout. If the heterogeneity of the meta-analysis was less than 50%, a fixed-effects model was applied, otherwise a random-effects model was utilized.

### Three-step SMR analysis at the gene level

We further employed SMR analysis [18] as a complementary analysis to prioritize causal variants mediated by gene expression. Furthermore, considering that DNAm located in promoters or enhancers usually influences the regulation of disease-associated target genes. We employed a three-step SMR to test whether the causal effects of identified druggable genes on gout are mediated by DNA methylation levels within the gene region. First, using SNPs as IVs, identified causal genes as exposure, and gout as outcome. Second, SNPs serve as IVs, causal genes-related DNA methylation QTLs (DNAms) as exposure, and gout as outcome. Finally, SNPs as IVs, DNAms as exposure, and identified causal genes as outcome. Summary data on mQTLs were provided by the meta-analysis of Brisbane Systems Genetics Study and Lothian Birth Cohorts (*n* = 1980) [18]. Meanwhile, HEIDI tests were performed on multiple SNPs in the same region [18]. SMR and HEIDI tests were performed based on SMR software (SMR v1.3.1). *P* < 0.05 was considered significant for SMR analysis. In the HEIDI test, *P* > 0.01 indicated that the gene-gout correlation was not driven by linkage disequilibrium. The final candidate signals were required to meet the following conditions: (1) pass the three-step SMR analysis (*P* < 0.05); (2) SNPs were suggestive of genome-wide significance in all eQTLs, mQTLs, and GWAS (*P* < 5 × 10^− 8^); (3) HEIDI derived P value > 0.01.

### SMR analysis at the protein level

We looked for evidence of causality at the protein level to strengthen the robustness of results. We obtained pqtl summary data of identified causal genes from five large-scale proteomic studies (Ferkingstad et al. [19]; Sun_1 et al. [20]; Sun_2 et al. [21]; Suhre et al. [22]; Folkersen et al. [23]) for SMR analysis and HEIDI testing. SMR derived P value < 0.05 and HEIDI derived P value > 0.01 are regarded as causal genes for gout established robust evidence of causal association at the protein level.

### Druggability evaluation

Phe-MR analysis identifies potential side effects and repurposing opportunities for drugs by examining causal relationships between genetic variants and various disease phenotypes. Prior to evaluating potential targets, we selected the GWAS for Phe-MR analysis from the UKB cohort (https://www.leelabsg.org/resources), which included 28 million SNPs across 1,403 disease traits in 408,961 white Britons. Phe-MR analysis methods consistent with previous MR analysis methods, including IVW, MR Egger, weighted median, RAPS, and others. P values were corrected by Bonferroni (*P* < 0.05/1403). To further assess the interaction between the identified gout causal genes and marketed drug targets, we searched the DrugBank database(https://go.drugbank.com/) for clinical drug targets that have been approved or are undergoing experiments for gout. Also, a PPI network between the gout causal genes and clinical drug targets for gout were established by using the STRING V12.0 database (https://cn.string-db.org/), setting the minimum required interaction score of 0.4 [24]. Causal genes with strong interactions with clinical drug targets were prioritised as having high clinical development potential. In addition, we utilize the DepMap data to assess whether genes are essential or not, with the results informing the prioritization of genes for drug targeting.

## Results

### MR analysis identified 8 druggable genes for gout

**Fig. 2:**
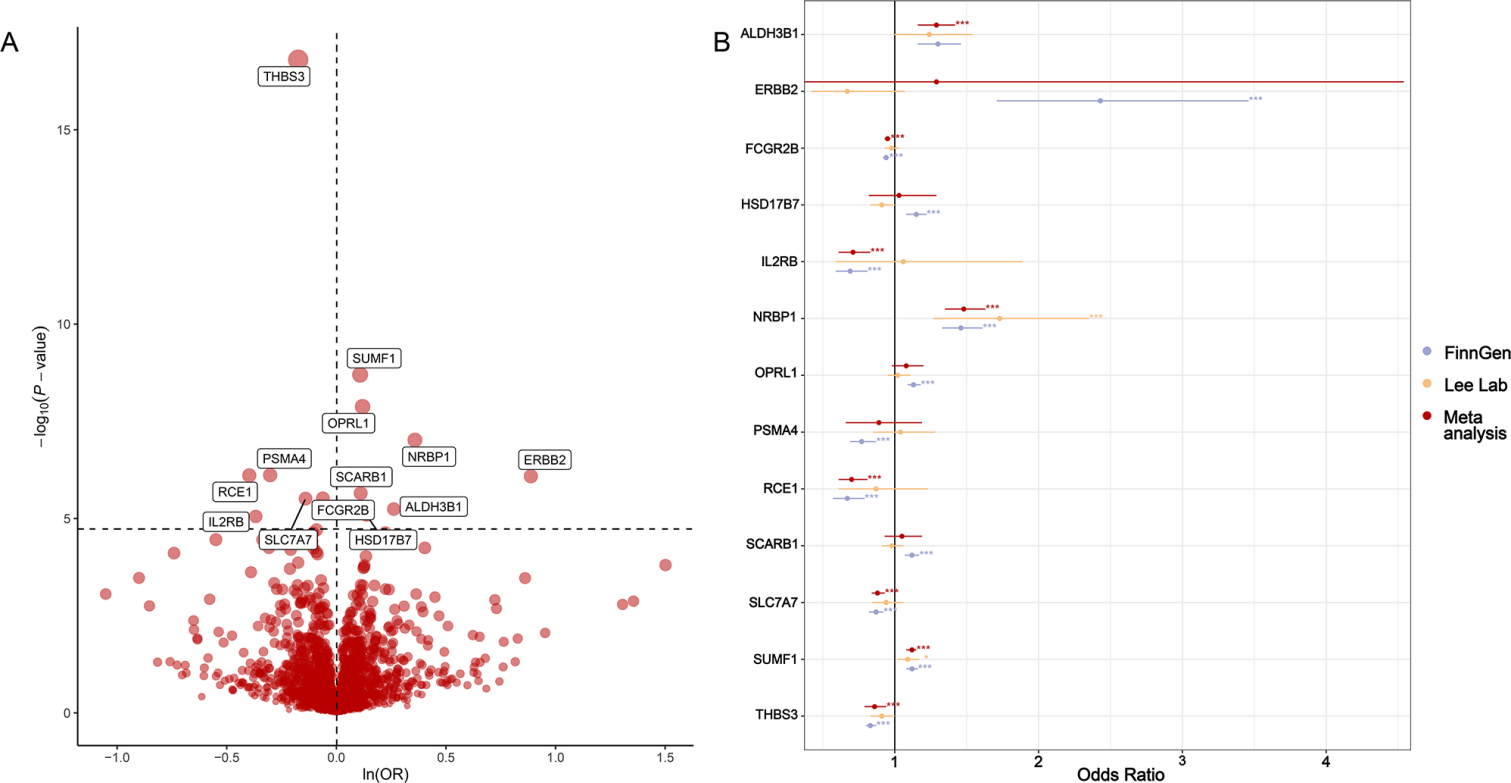
Druggable genes with significant causal association with gout. (**A**) Initial identification of 13 drug availability genes with significant causal association with gout based on FinnGenR10 consortium. (**B**) Based on the replication analysis of Lee Lab cohort, and meta-analysis, 8 drug candidate targets for gout were finally established

A total of 2633 drug availability genes were assessed for causal association with gout in the primary MR analysis using the Gout GWAS of the FinnGenR10 (Table S2). After Bonferroni adjustment, q-values and BH correction, 13 drug availability genes were preliminarily identified as having a significant causal association with gout based on the IVW method (Fig. 2A, Figure S1 and Table S3). Outliers were identified and excluded using IVW Radial MR (Table S4). No heterogeneity (Cochran Q derived *P* > 0.05) or pleiotropy (MR Egger intercept derived *P* > 0.05) was probed in sensitivity analysis (Table S5). LOO analysis also suggested that MR estimates were not driven by a single SNP (Figure S2). In Steiger directionality tests, there’s no reverse causal association between availability genes and gout (Table S5). Replication analysis based on MR estimates generated by the IVW method in the Lee Lab cohort, and meta-analysis results (Table S6 and Fig. 2B) indicated that the significant causal association of 8 druggable genes comprising 3 risk genes (ALDH3B1, NRBP1, SUMF1) and 5 protective genes (FCGR2B, IL2RB, RCE1, SLC7A7, THBS3) with gout was robust (P_meta−analysis_ < 0.05). Additionally, we further performed detailed functional annotation and pathway analysis of these eight genes (Table S7 and S8).

### Putative gout causal genes mediated by blood methylation regulation on gene expression

**Fig. 3:**
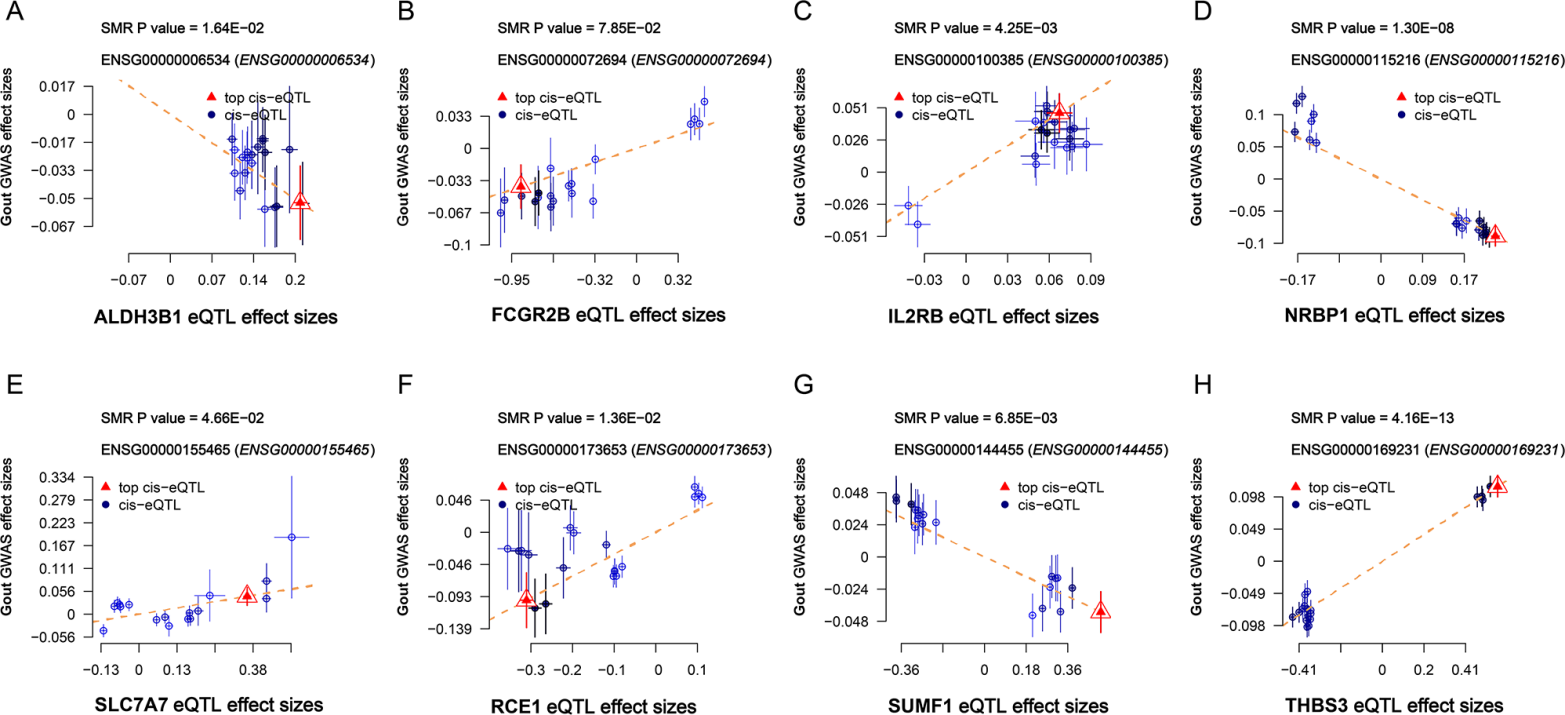
Results of SMR analysis of eight candidate gout-causal genes

We performed SMR analysis and HEIDI testing on the 8 identified gout causal genes to exclude LD interference (Fig. 3 and Figure S3-S10). Notably, all 8 genes passed SMR analysis (*P* < 0.05) and 7 genes passed the HEIDI test (*P* > 0.01) (Table S9). The causal association of NRBP1 with gout may be weakly biased by LD (P_HEIDI_ < 0.01). At the methylation sites in eight gene regions, we only found that genetically predicted each SD increase in methylation of cg25402137 at the ALDH3B1 was associated with a reduced risk of gout (β = -0. 08, 95% confidence interval [CI]: -0.16-0.00 *P* = 0.044), while Each SD increase in methylation levels of cg02999224 (β = 0. 18, 95% CI: 0.03–0.34, *P* = 0.022) and cg16465430 (β = 0.07, 95% CI: 0.00-0.14, *P* = 0.048), located in the SLC7A7 gene region, was linked to increased gout risk (Table S10). SMR analysis of DNA methylation levels and gout causal gene expression identified seven DNA methylation sites (ALDH3B1: cg18412889, cg23121335, cg25402137; IL2RB: cg25246692, cg11558856; NRBP1: cg15478930; SUMF1: (cg24840601) modifications may regulate ALDH3B1, IL2RB, NRBP1 and SUMF1 expression (Table S11). Combining the results of the three-step SMR analysis, we found that genetically predicted elevated levels of CpG site cg25402137 located in ALDH3B1, reduced the risk of gout by down-regulating ALDH3B1 expression levels.

### SMR analysis at the protein level

At the protein level, SMR analysis revealed significant causal associations between IL2RB, NRBP1, SUMF1, and THBS3 levels and the risk of gout (Table S12). However, only NRBP1 and SUMF1 were consistent with the direction of the effect of gout-producing SMR estimates at the protein and transcriptome levels.

### Phenome-wide MR analysis

**Fig. 4:**
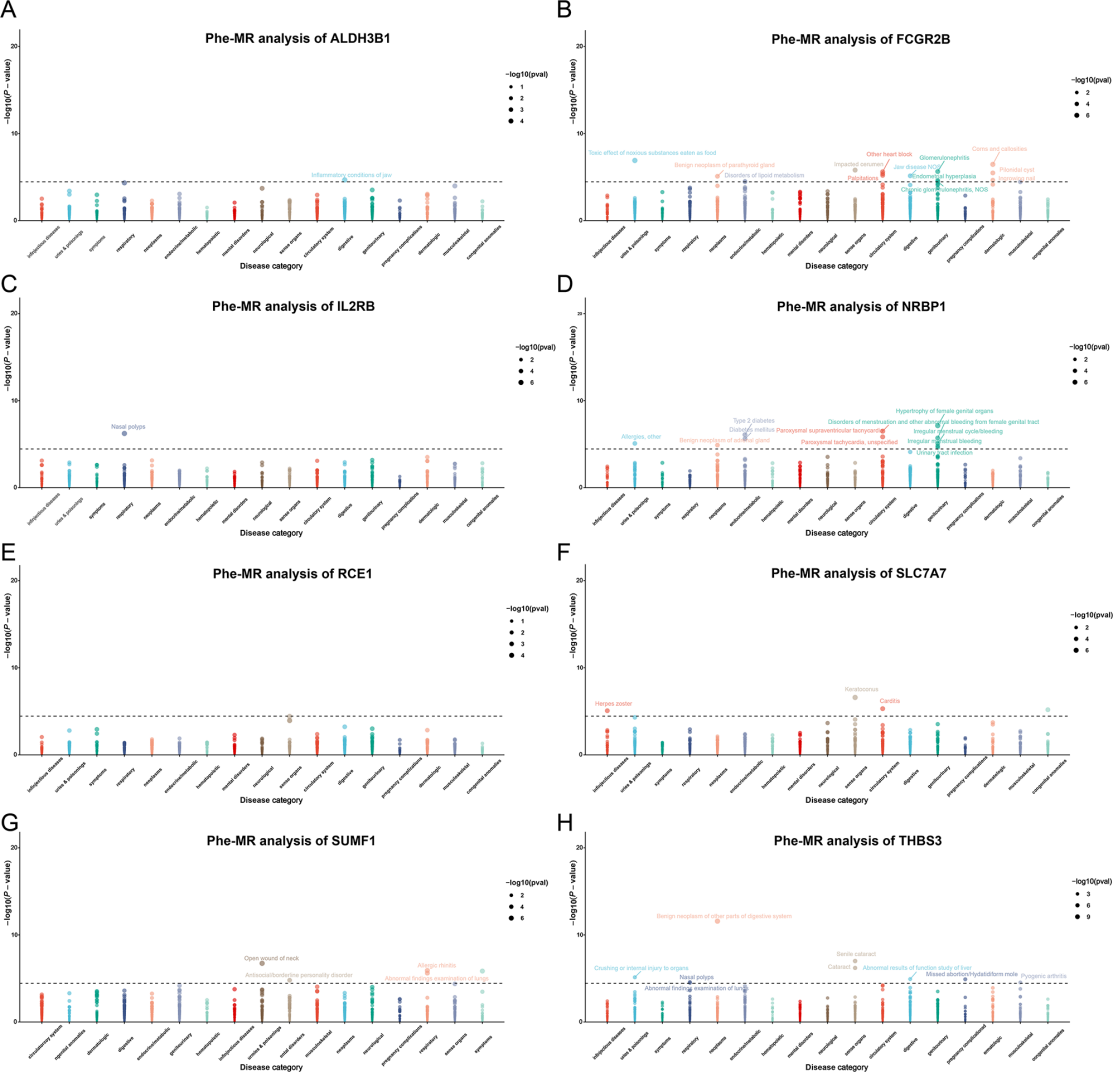
Phe-MR results of the 8 candidate drug targets

We conducted a comprehensive analysis for 1403 disease traits to fully characterize the information on identified drug targets. In the Phe-MR analysis, a total of 45 relevant traits reached the significance threshold of *P* < 0.05/1403 based on the IVW method (Fig. 4 and Table S13). Briefly, for instance, targeting ALDH3B1 may be beneficial for 1 digestive system disease (Inflammatory conditions of jaw). Targeting SLC7A7 may have adverse effects on the circulatory system (Carditis) and congenital anomalies (Cardiac shunt/ heart septal defect), while it may be beneficial for infectious diseases (Herpes zoster) and sense organs (Keratoconus) and sense organs (Keratoconus). Whereas targeting NRBP1 will be beneficial to endocrine/metabolic system but may cause multi-system deleterious effects such as circulatory system, respiratory and injuries & poisonings, genitourinary. We additionally performed MR scans of gout in 1403 diseases, however, no significant association with gout was found (Table S14).

### Druggability evaluation

**Fig. 5:**
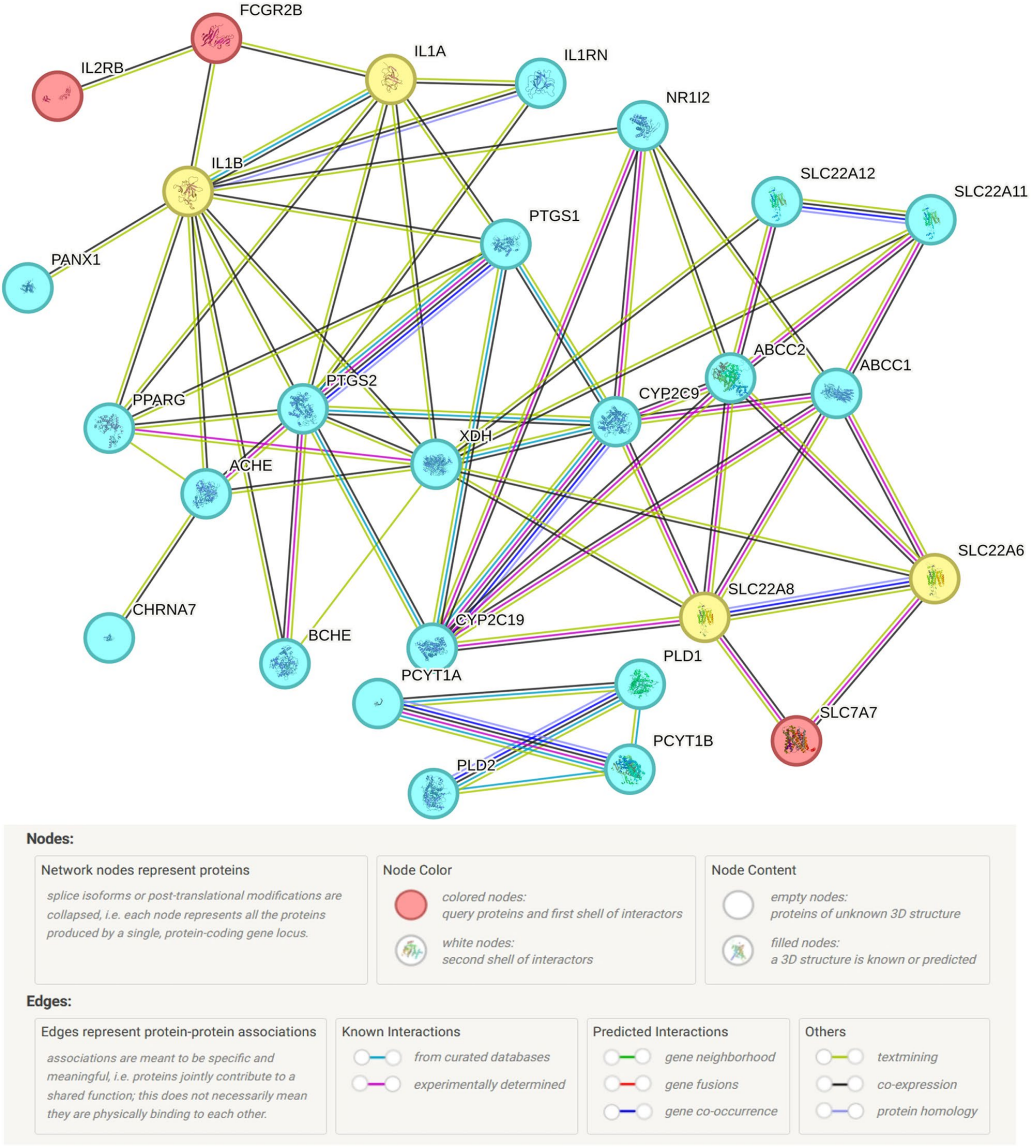
PPI networks between gout causal genes and marketed drug targets

In the DrugBank database, we searched for information on currently approved drugs or drug targets being tested for gout (Table S15). By PPI analysis we found evidence based on experimental determined that the identified gout causal gene SLC7A7 was strongly correlated with the targets SLC22A6 and SLC22A8 of probenecid (Fig. 5). Additionally, FCGR2B was co-expressed with the targets of Rilonacept, IL1B and IL1A. To further refine the draggability assessment, we assessed the essentiality of the genes using the DepMap dataset. The analysis showed that NRBP1 was an essential gene (mean dependence score: -0.9385), indicating that it has a critical role in cell survival and targeting it may be risky. The other genes (ALDH3B1, FCGR2B, IL2RB, RCE1, SLC7A7, SUMF1, and THBS3) were non-essential, suggesting that these genes have high targeting potential and low risk (Table S16 and Figure S11).

## Discussion

Within this study, we performed a three-step SMR analysis to explore genetically predicted gout-associated drug targets, as well as the association of gene methylation expression levels with drug targets. To the best of our knowledge, this is the first MR study to date that applies multi-omics GWAS data to explore potential drug targets of gout. Ultimately, eight genes were identified as possible drug targets in gout, including three risk genes (ALDH3B1, NRBP1, SUMF1) and three protective genes (FCGR2B, RCE1, SLC7A7). For these eight genes, GO annotation revealed a significant enrichment of genes involved in biological processes such as immune response, inflammatory response, cytokine-mediated and purine metabolism, which are associated with the pathophysiology of gout. In addition, KEGG pathway analysis identified several key pathways, including Th1 and Th2 cell differentiation, Th17 cell differentiation, and the JAK-STAT signaling pathway, which are closely related to the inflammatory response in gout. Given the conflicting directions of IL2RB and THBS3 effects at the gene and protein levels, further exploration of their roles in gout is warranted. NRBP1 and SUMF1 have been validated at both the gene and protein levels with a high level of evidence, and they may have a greater potential for exploitation. In druggability evaluation, we found that SLC7A7 may have greater potential for gout drug development, whereas targeting NRBP1 needs to be carefully considered.

A previous GWAS study identified NRBP1 as a risk gene for gout and was shown to have elevated expression in human peripheral blood single nucleated cells from gout patients [25]. This is consistent with our findings. This study found that hypomethylation of the NRBP1 promoter region reduces the binding of NRBP1 to the transcription factor TFAP2A, which leads to elevated levels of NRBP1 and may contribute to the development of gout. In addition, NRBP1 is associated with sodium and albumin excretion and/or metabolism and may also be involved in the pathogenesis of gout [26]. In Zhang et al.‘s study, overexpression of NRBP1 significantly decreased the expression of uric acid transporter proteins GLUT9 and URAT1, whereas NRBP1 inhibition significantly increased the expression of ABCG2 and promoted uric acid excretion via the kidney in HK-2 cells [27]. NRBP1 is involved in the important process of urate homeostasis, and drug development against NRBP1 can improve uric acid excretion or inhibit renal reabsorption of uric acid, which may be a major effective way to prevent and treat gout. However, NRBP1 plays a critical role in cell survival. If NRBP1 is inhibited as a potential drug target, normal cells may be severely affected, which may increase the risk of drug development. Also, effects on the circulatory, genitourinary, injury and toxicity, and tumor systems need to be addressed simultaneously. Therefore, NRBP1 should be carefully considered in drug target prioritization assessment.

SUMF1 is a key control factor for maintaining sulfate esterase activity and is responsible for encoding the formylglycine-generating enzyme that converts cysteine to formylglycine to accomplish post-translational modifications to sulfate esterase [28]. Although SUMF1 and its sulfate esterase targets have not been studied in relation to gout for the time being, mutations in SUMF1 and its sulfate esterase targets lead to a variety of human diseases, especially lysosomal storage diseases [29]. Lysosomal function is concerned with MSU crystal-induced activation of NLRP3 inflammatory vesicles [30], and the specific mechanism of effect needs to be further explored.

In the druggability assessment, SLC7A7 and FCGR2B were validated, and both were significantly associated with relevant targets of the currently commercially available gout drugs Probenecid and Rilonacept, respectively. Although no relevant target drugs are available at present, these evidences suggest that they may have higher development potential in gout. SLC7A7 deletion causes lysine-urinary protein intolerance (LPI). LPI is characterized by dysfunction of the urea cycle and renal disorders, which may lead to an imbalance of urate accumulation [31]. Additionally, SLC7A7 deletion or LPI renders the y + LAT1 transporter protein dysfunctional, which is essential for the exocytosis of arginine, lysine, and ornithine [32, 33]. In the assessment of druggability, SLC7A7 was strongly associated with SLC22A6 and SLC22A8, which are the targets of current Probenecid drugs, and mainly inhibit the organic anion transporter proteins OAT1 (SLC22A6) and OAT3 (SLC22A8) to reduce uric acid reabsorption and increase uric acid excretion. Previous studies have shown that FCGR2B deficiency activates autoreactive T cells to develop arthritis [34]. Moreover, FCGR2B deficiency counteracts Hc encoding complement C5 action to accelerate the rapid progression of arthritis [35]. This implies the existence of a protective effect of FCGR2B against arthritis. From the assessment of the pharmacogenetic properties, FCGR2B connects IL-1β, IL-1α and IL2RB and seems to be closely linked to the interleukin family. The study by Stefanie et al. also supports that FCGR2B variant allele reduces serum IL-6 expression in patients with rheumatoid arthritis [36]. There are no relevant target drugs available, and further in vitro binding assays could be performed to confirm direct interactions between these targets and drugs.

At the methylation level, genetically predicted methylation of cg25402137 reduces the risk of gout development by down-regulating the expression level of ALDH3B1. ALDH3B1 is up-regulated in a wide range of human tumors, possibly as a result of resistance to cellular oxidative stress [37]. Also, Wu et al. [38] found that elevated ALDH3B1 expression reduced lipid peroxidation-induced damage to interfollicular epidermal cells. It appears that ALDH3B1 has some defense against oxidative stress. RCE1 shows correlation with tumor progression, e.g., overexpression is positively correlated with prostate cancer [39], and down-regulation predicts poor prognosis in hepatocellular carcinoma [40, 41]. Although no relevance for direct targeting of gout has been demonstrated for the time being, they remain the more promising target, as no predicted adverse effects were seen in the Phe-MR analysis.

Notably, IL2RB and THBS3 have contradictory directions of effect on gout at the protein and transcriptional levels. A study found that THBS3 mediated 19.23% of the association between hyperuricemia and coronary heart disease [42]. In a study by Teng et al. [43], the C allele of the rs12411216 genotype in the THBS3 gene region was associated with lower uric acid levels and risk of gout, which may be a mechanism by which THBS3 is involved in and protects against the development of gout. This is consistent with our findings at the gene level, identifying THBS3 as a protective gene. IL2RB has mainly been described to play a role in immune harmony involving immune tolerance to regulatory T cells (Treg) [44]. Gout is also recognized as an autoinflammatory disease, and it has been found that elevated levels of the serum cytokine IL-2 may play a key role in the pathogenesis of gout, and restoration of Th17/Treg homeostasis may be a potential therapeutic direction [45]. Nevertheless, there are limited studies on THBS3 and IL2RB in gout and more studies are needed to clarify.

This work has the following strengths. In this work, we used a two-stage MR design to systematically investigate the association between 2633 druggable genes and gout risk. The design has the advantages of large sample size, rich genome coverage, reverse causality and minimal risk of confounding bias. The robustness of the findings was confirmed by the consistency of the results of multiple rigorous analyses. Additional evidence from Phe-MR analyses and druggability evaluations provided an assessment of the side effects and druggability of the gout candidate targets. Admittedly, some limitations of this study should also be considered. First, the current analysis is limited to European populations. Extension of these findings to other ancestries requires further confirmation. Second, owing to the lack of complete pooled data at the protein level, some of the homozygous genes could not be analyzed by SMR to verify the reliability of the MR findings. Also, further mechanistic studies are needed to reveal the “driver” and “downstream” genes involved in the pathogenesis and development of gout.

## Conclusions

This study identified eight potential drug-targeting genes for gout, among which NRBP1 and SUMF1 have a greater potential for development. This work provides promising targets for the development of gout-related therapeutic agents. More studies are still needed in the future to evaluate their roles in gout to validate the current findings.

## Electronic supplementary material

Below is the link to the electronic supplementary material.

Supplementary Material 1

Supplementary Material 2

Supplementary Material 3

## Data Availability

No datasets were generated or analysed during the current study.
