# Supplementary figures and images for: Genetic insights into therapeutic targets for gout: evidence from a multi-omics mendelian randomization study
Source: Hereditas. 2024 Dec 30;161:56. doi: 10.1186/s41065-024-00362-8 (PMC11684267; doi:10.1186/s41065-024-00362-8)

QQ Plot of Druggable genes with significant causal association with gout

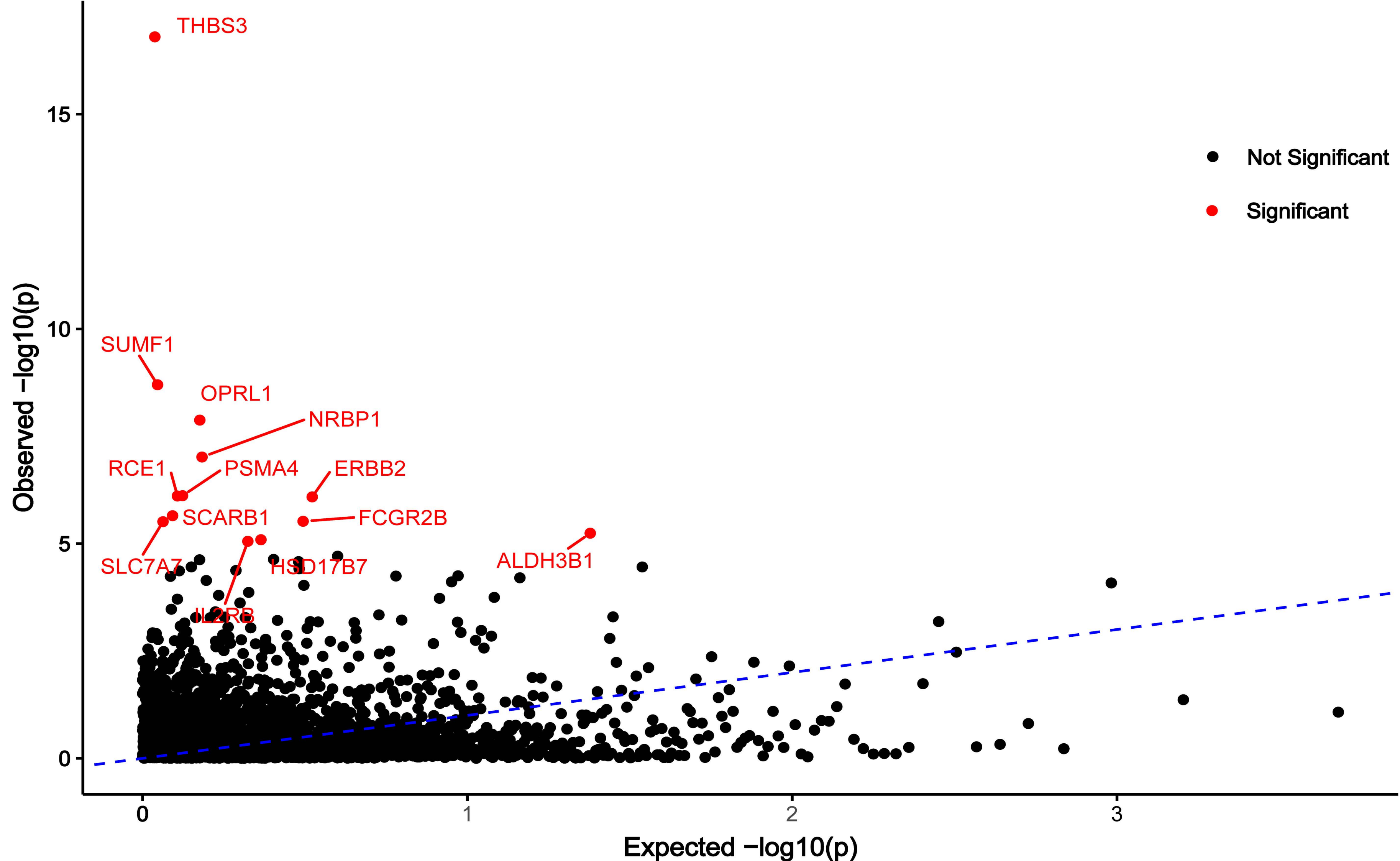

Supplement: Supplementary file 2 — Supplementary Material 2 [file 41065_2024_362_MOESM2_ESM.zip › Figure S1.pdf]

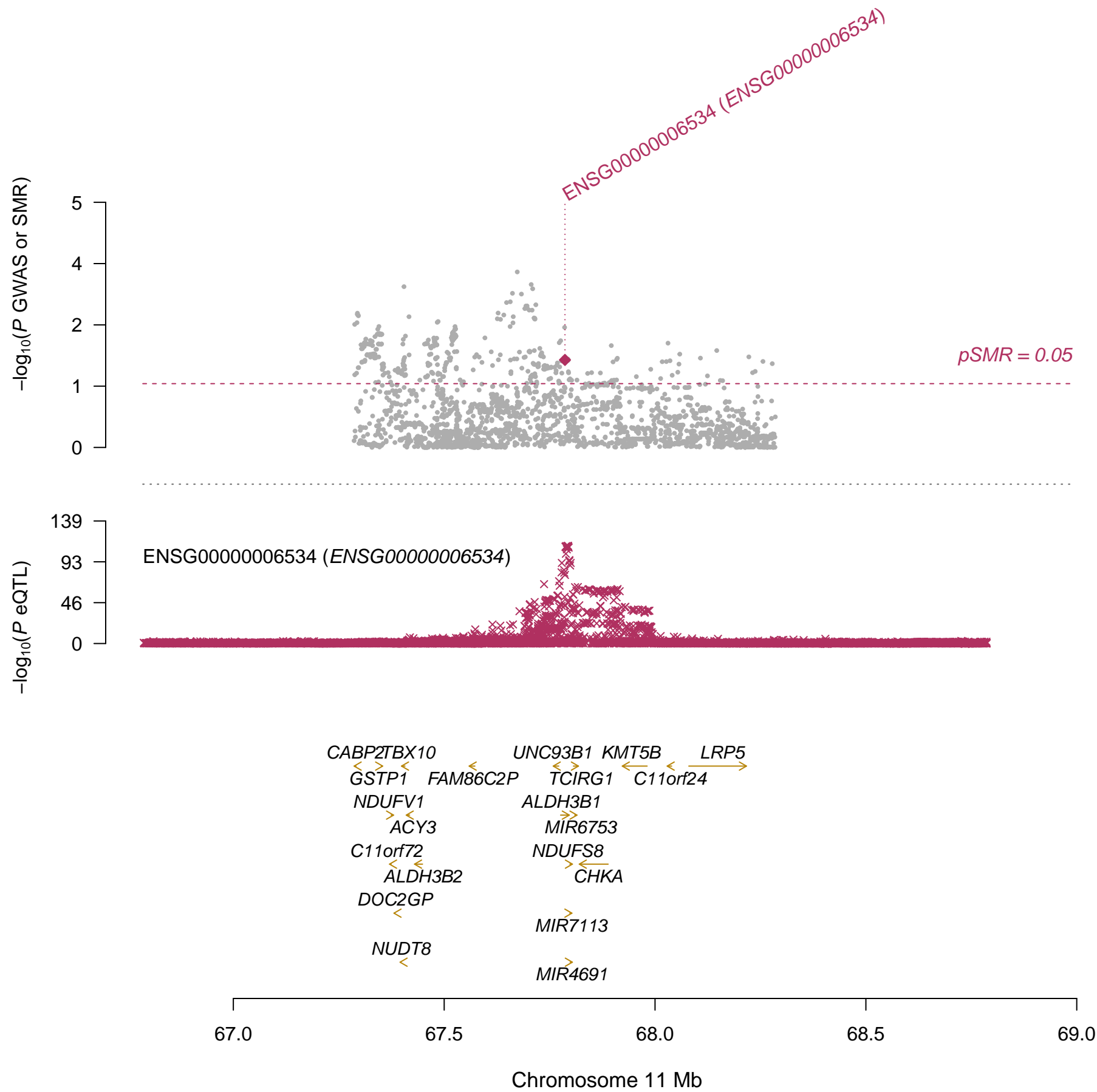

Supplement: Supplementary file 2 — Supplementary Material 2 [file 41065_2024_362_MOESM2_ESM.zip › Figure S10 ALDH3B1.pdf]

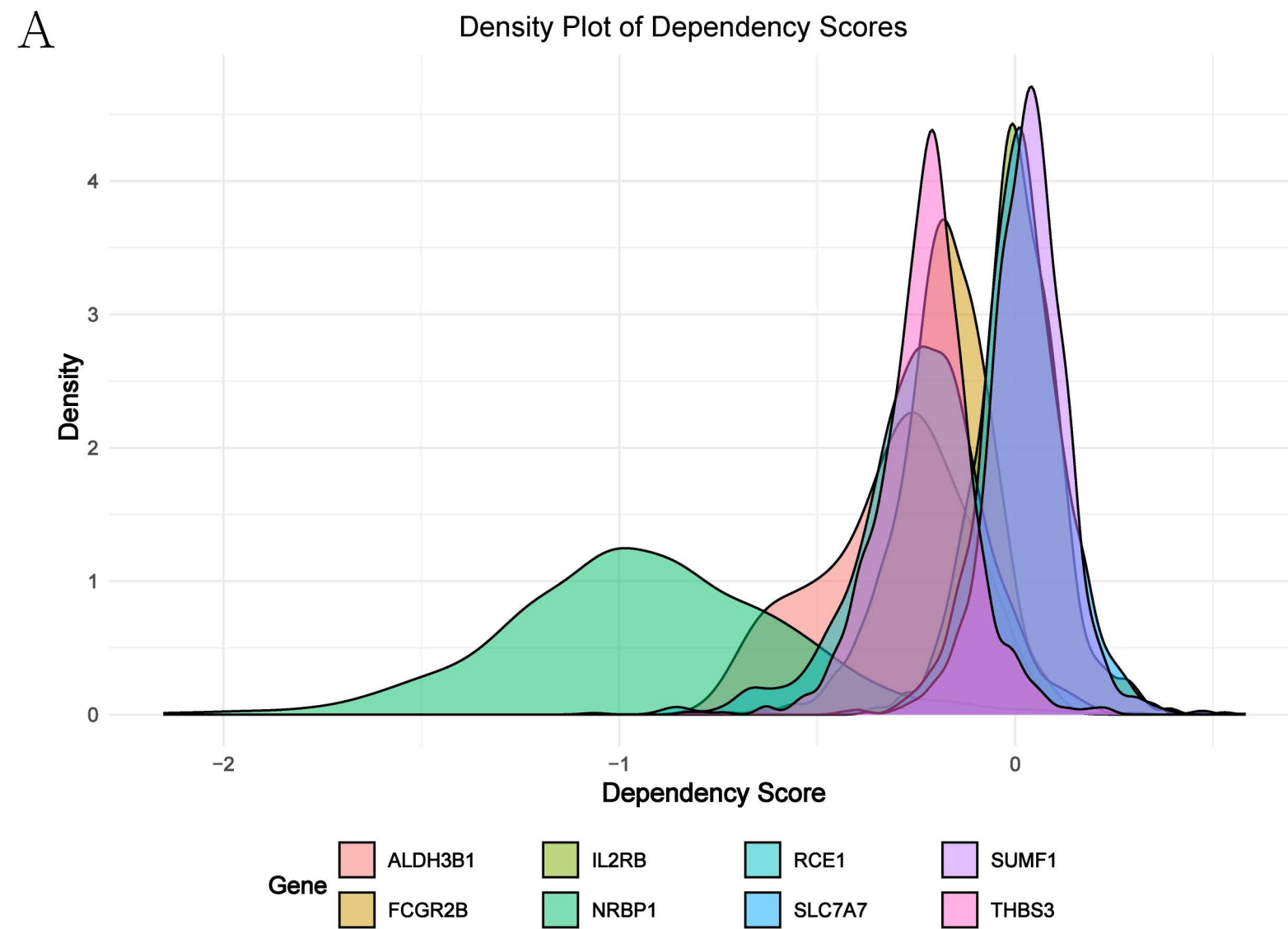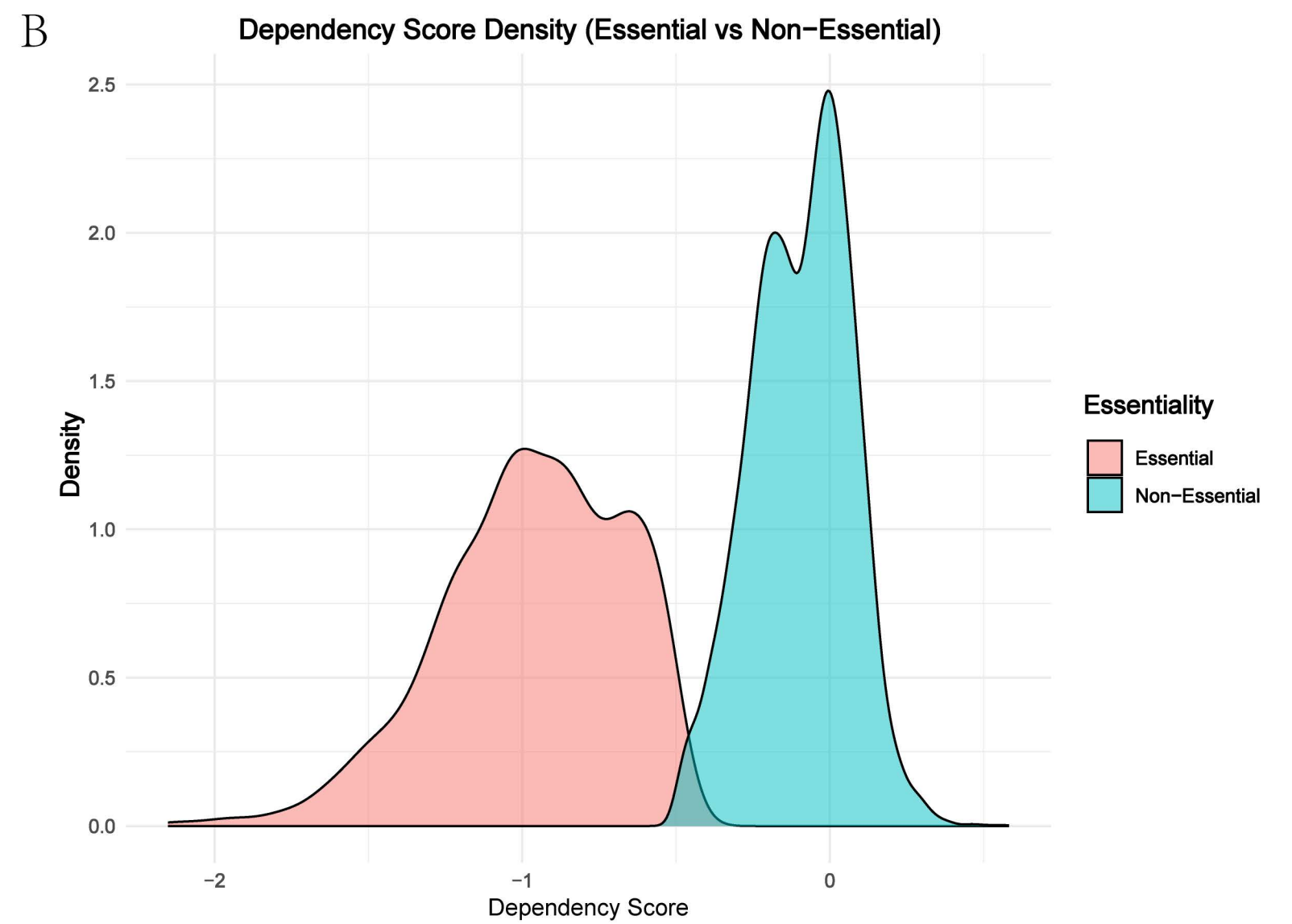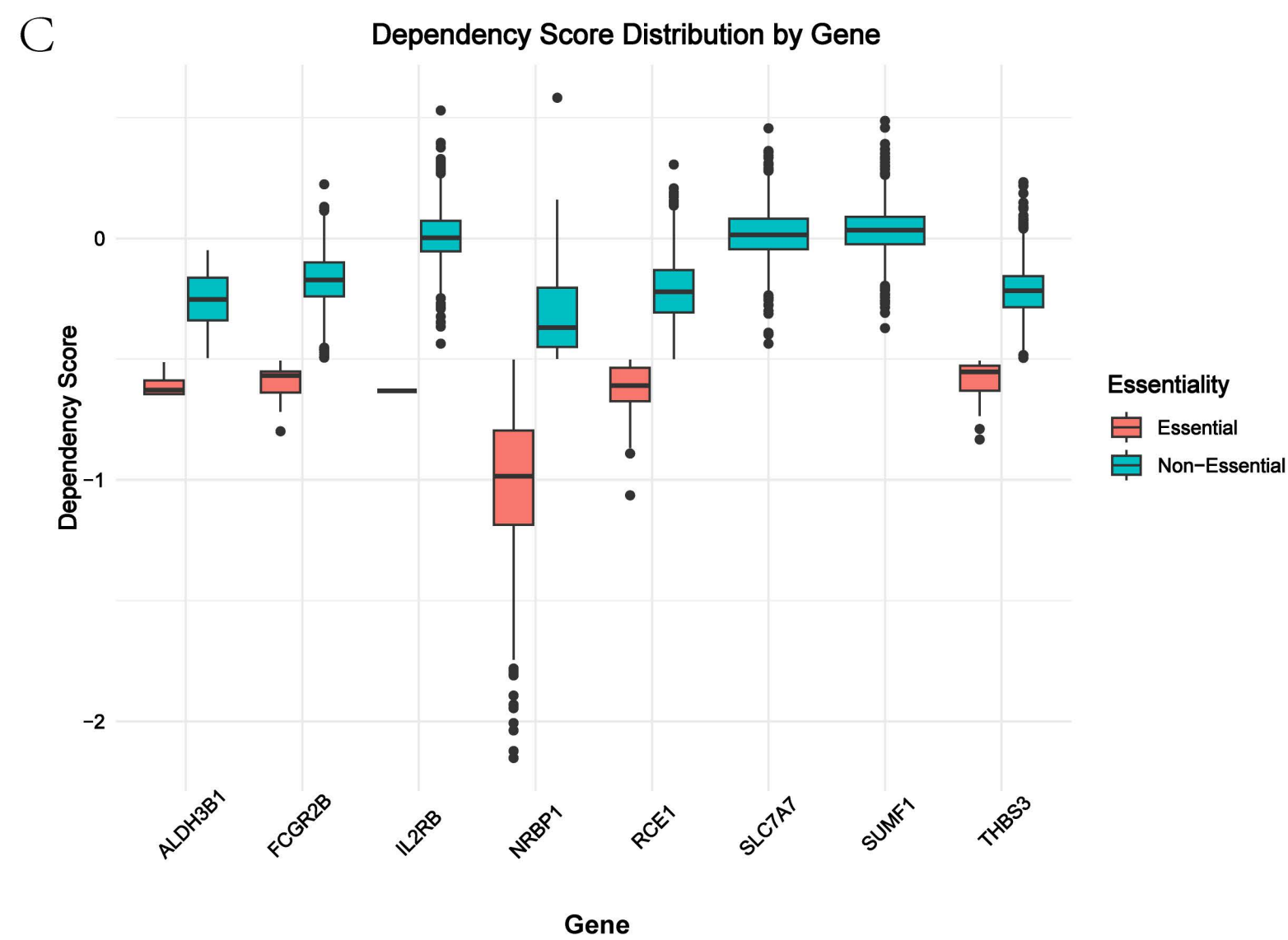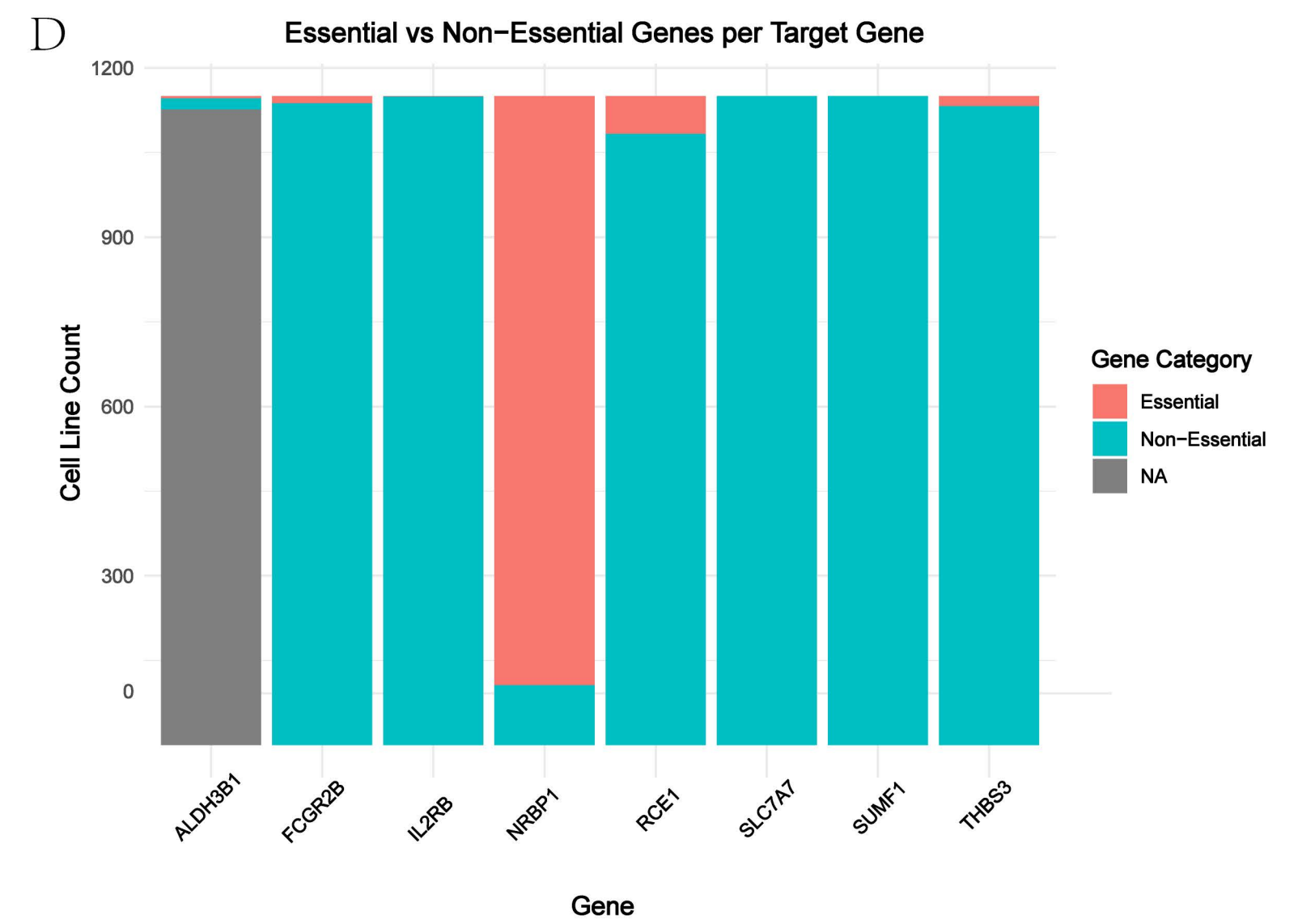

Supplement: Supplementary file 2 — Supplementary Material 2 [file 41065_2024_362_MOESM2_ESM.zip › Figure S11.pdf]

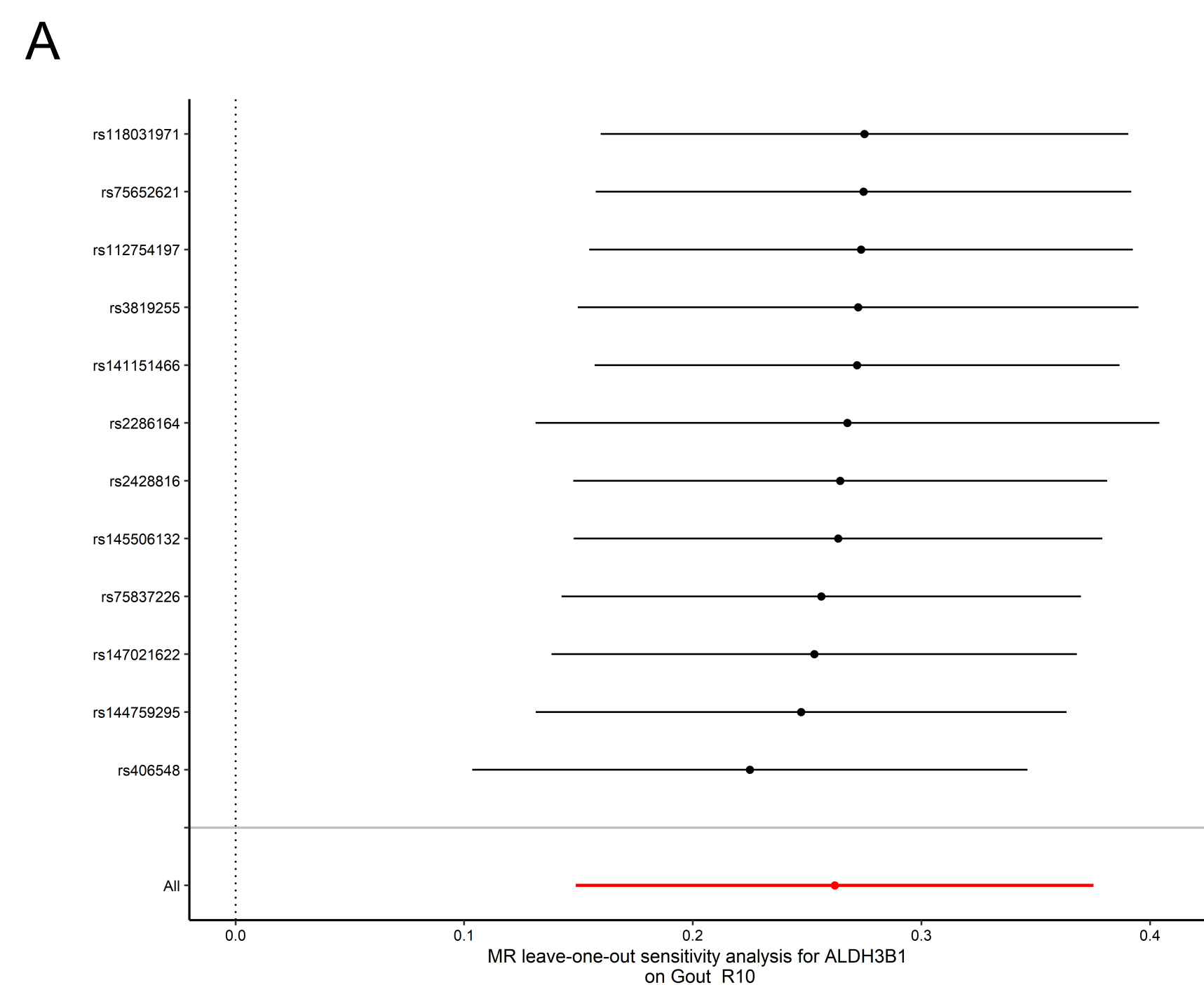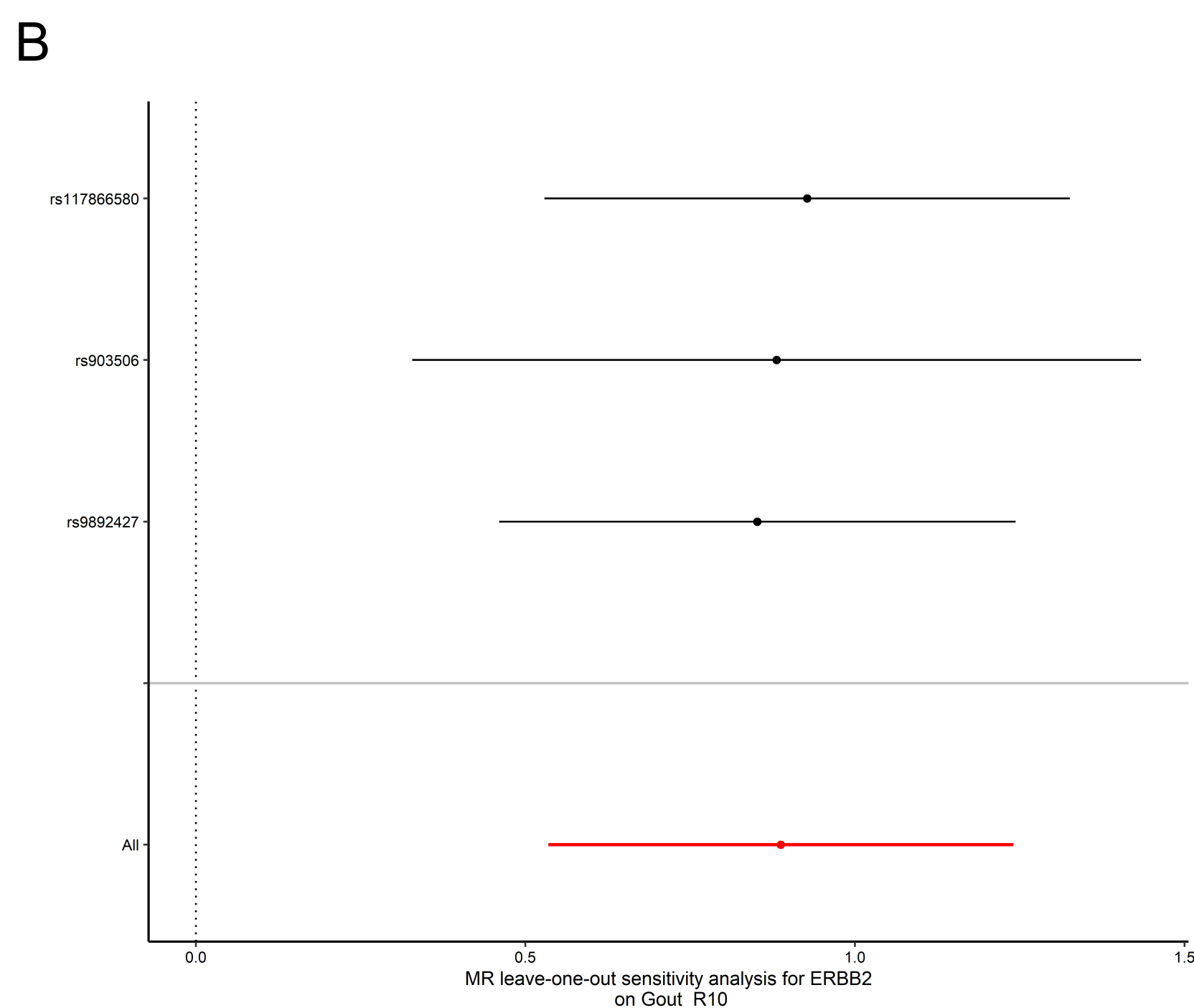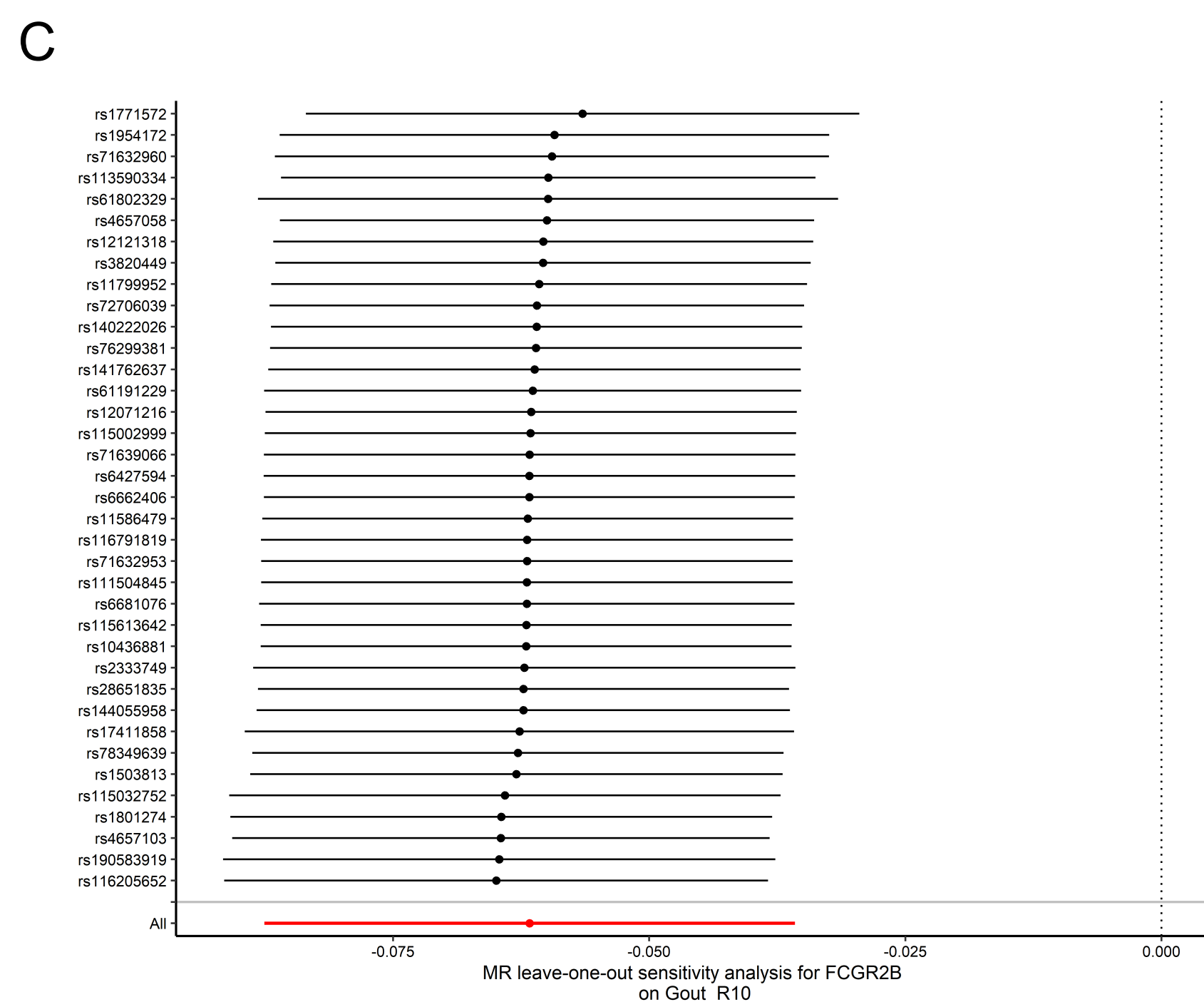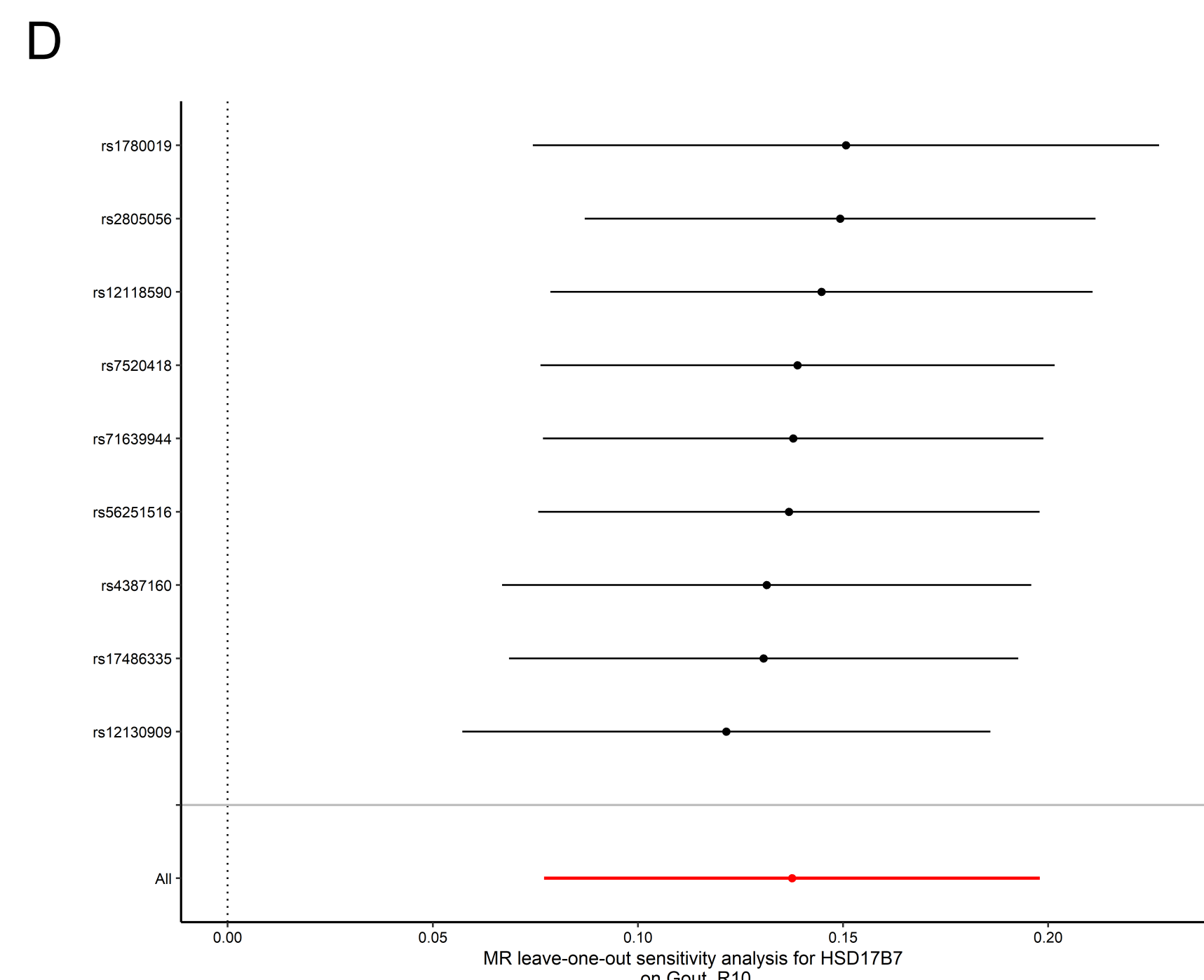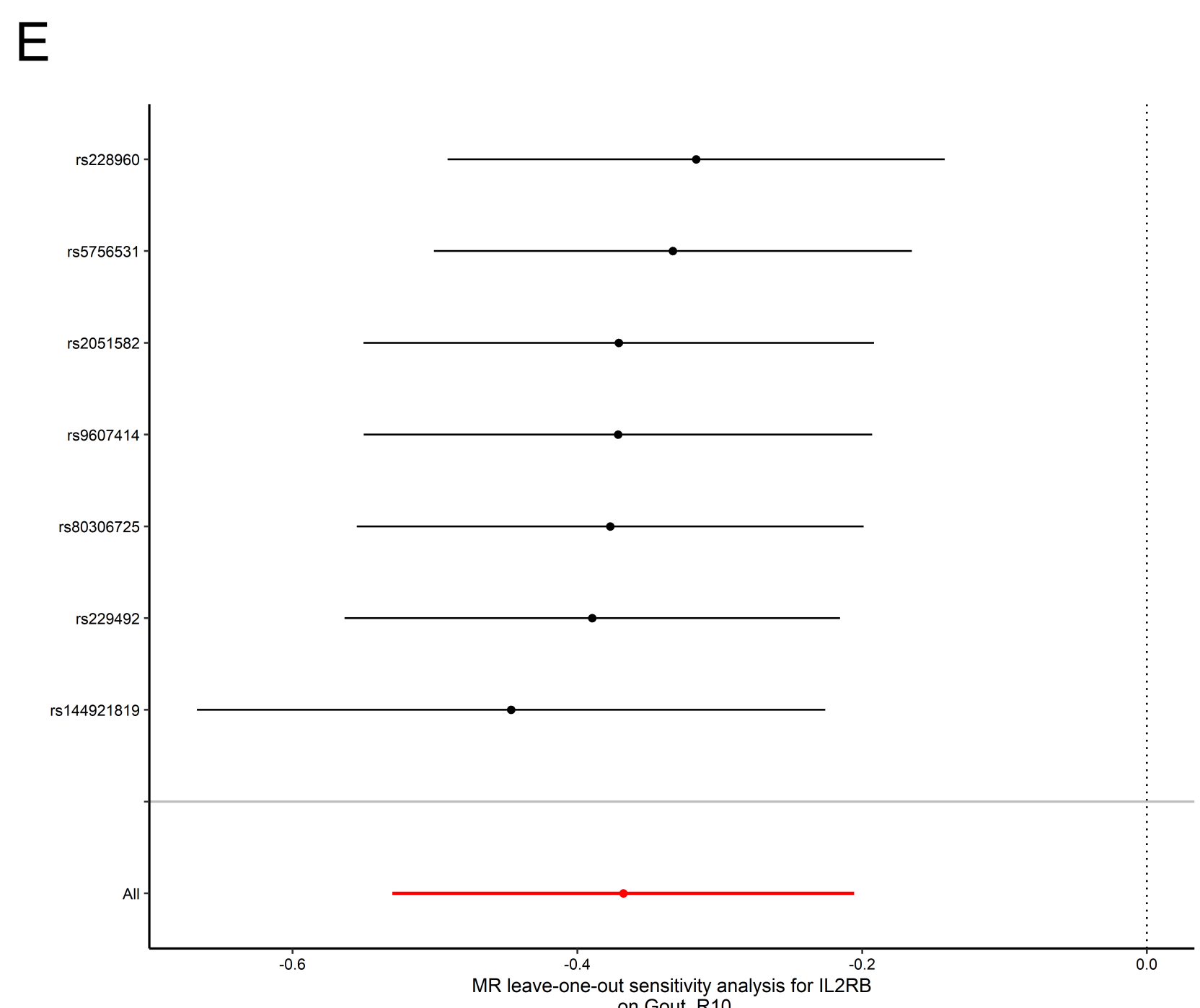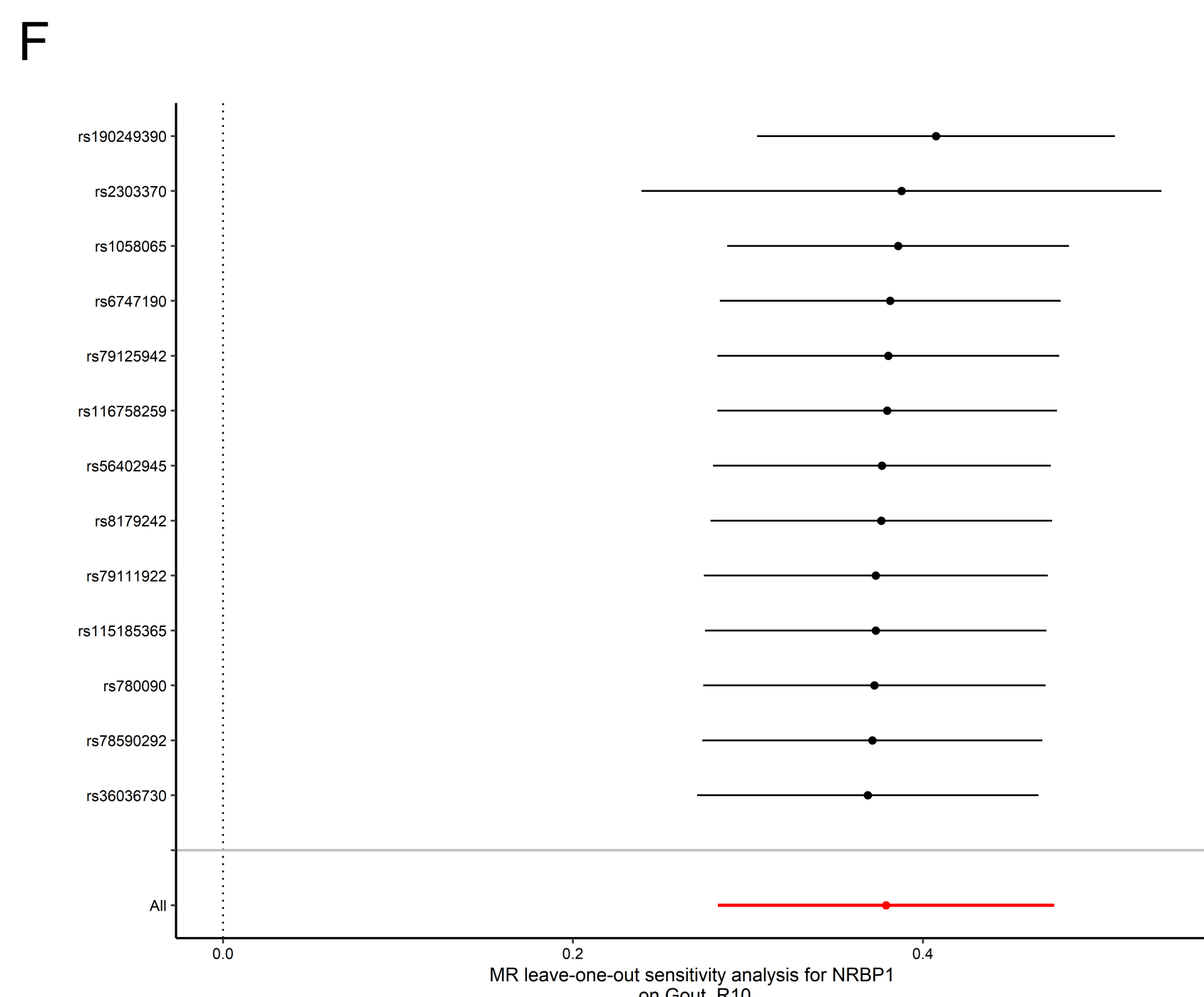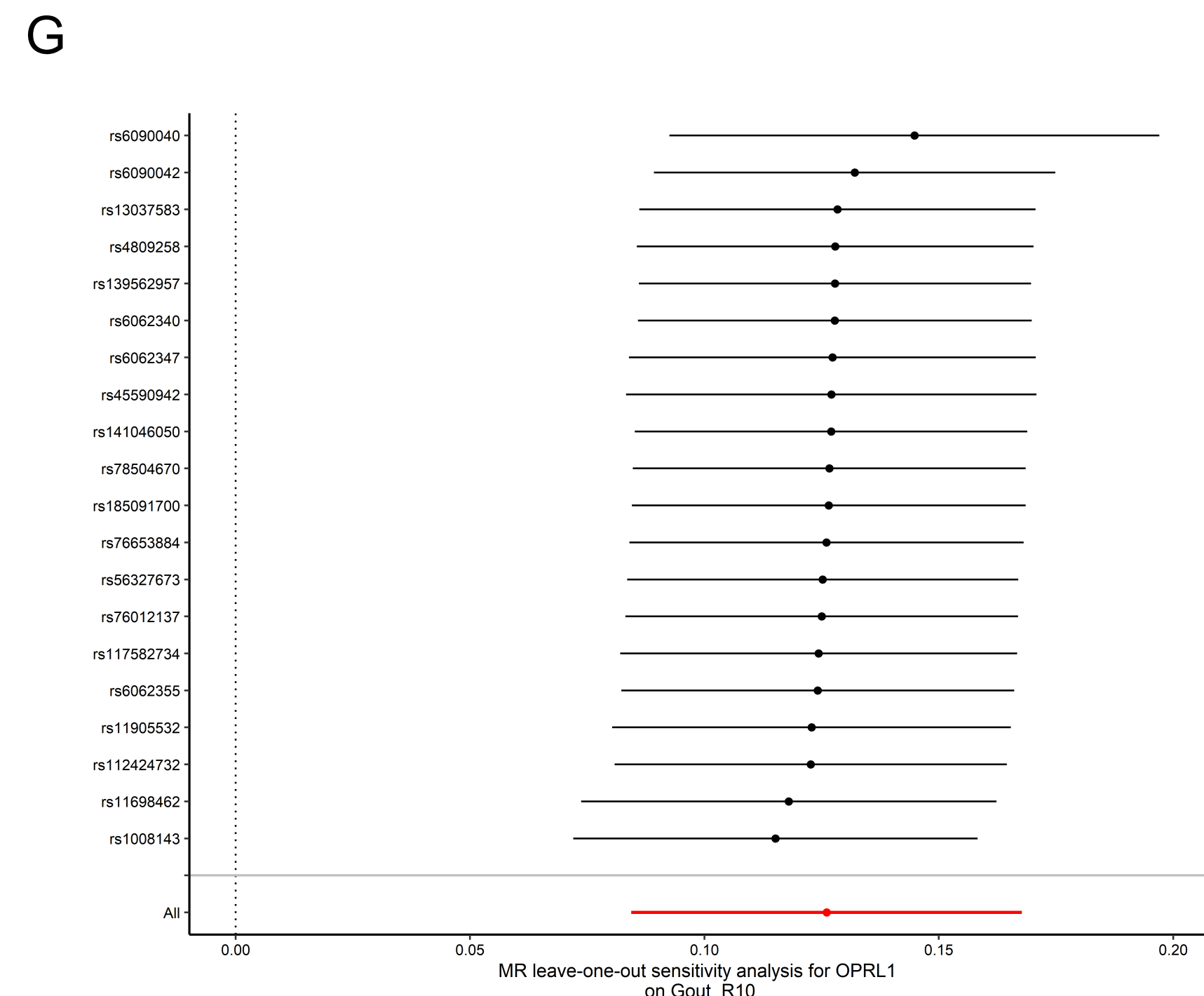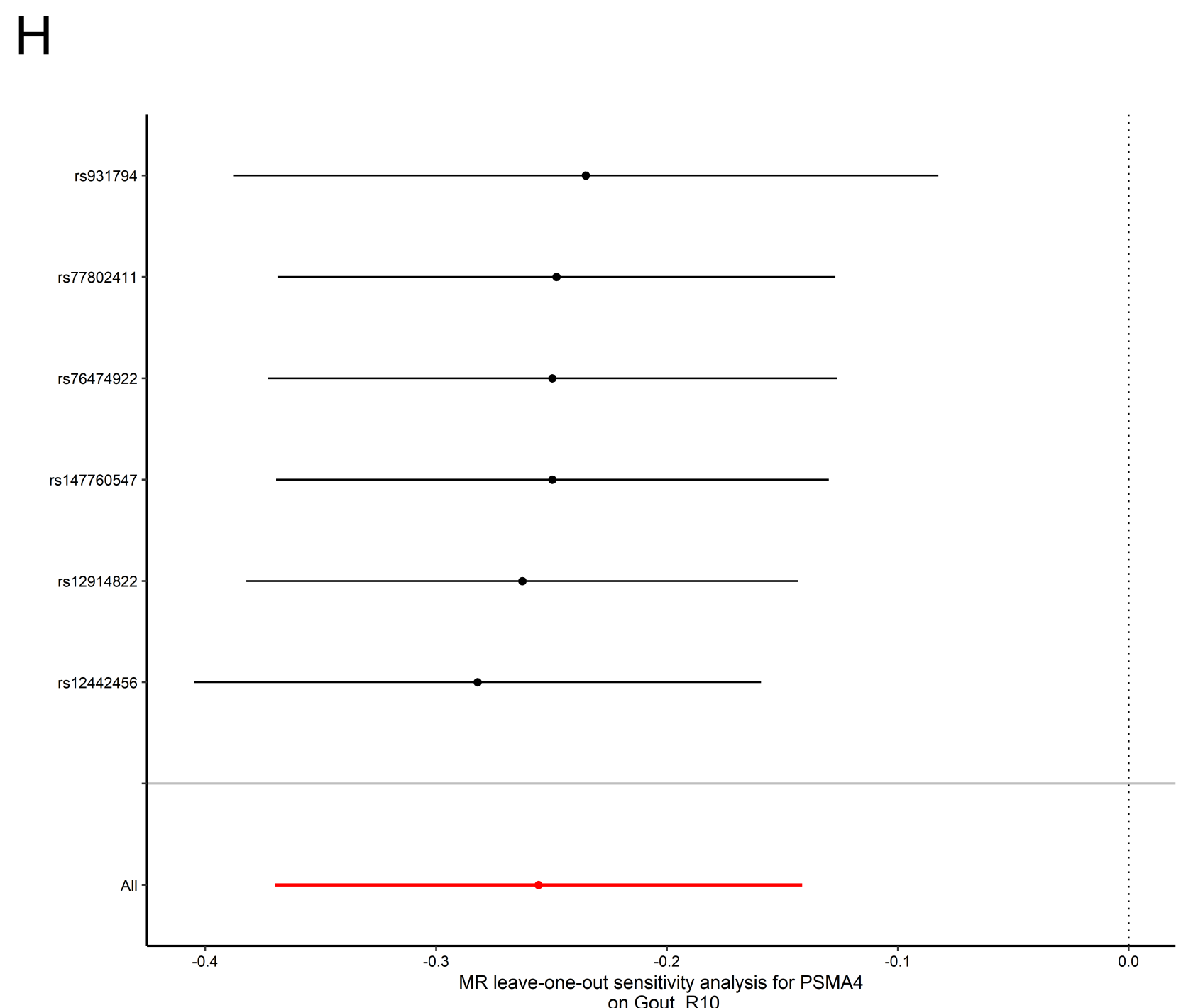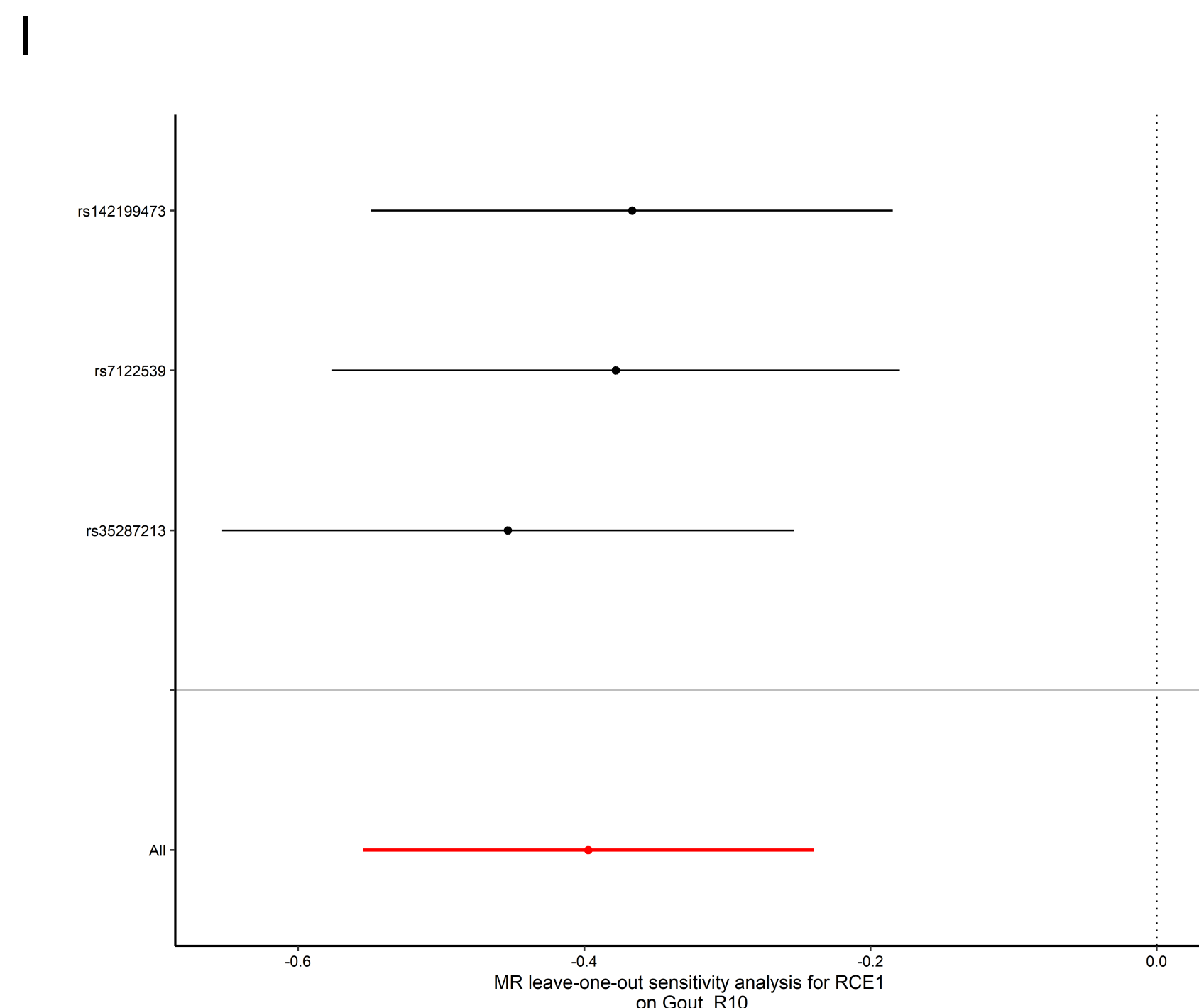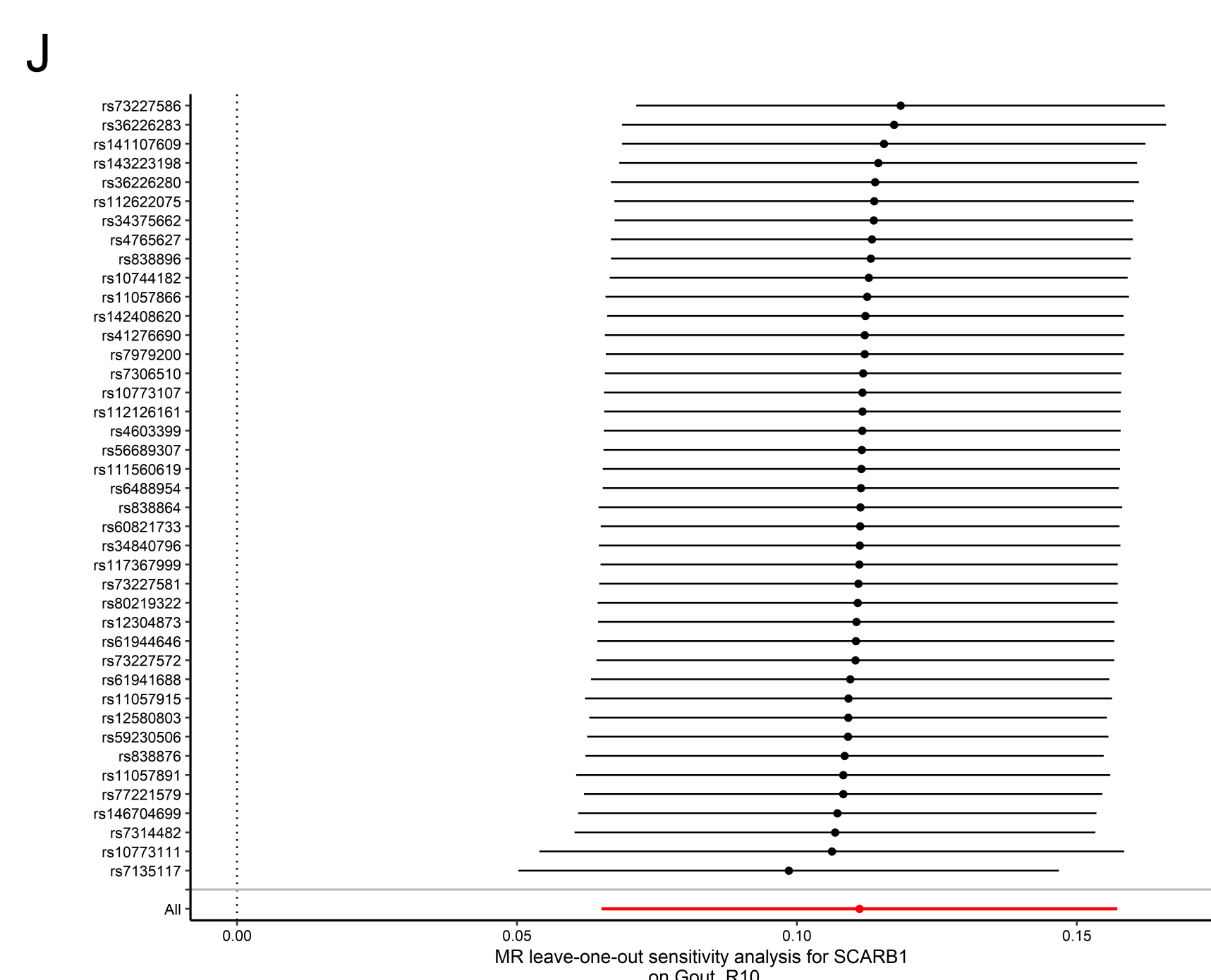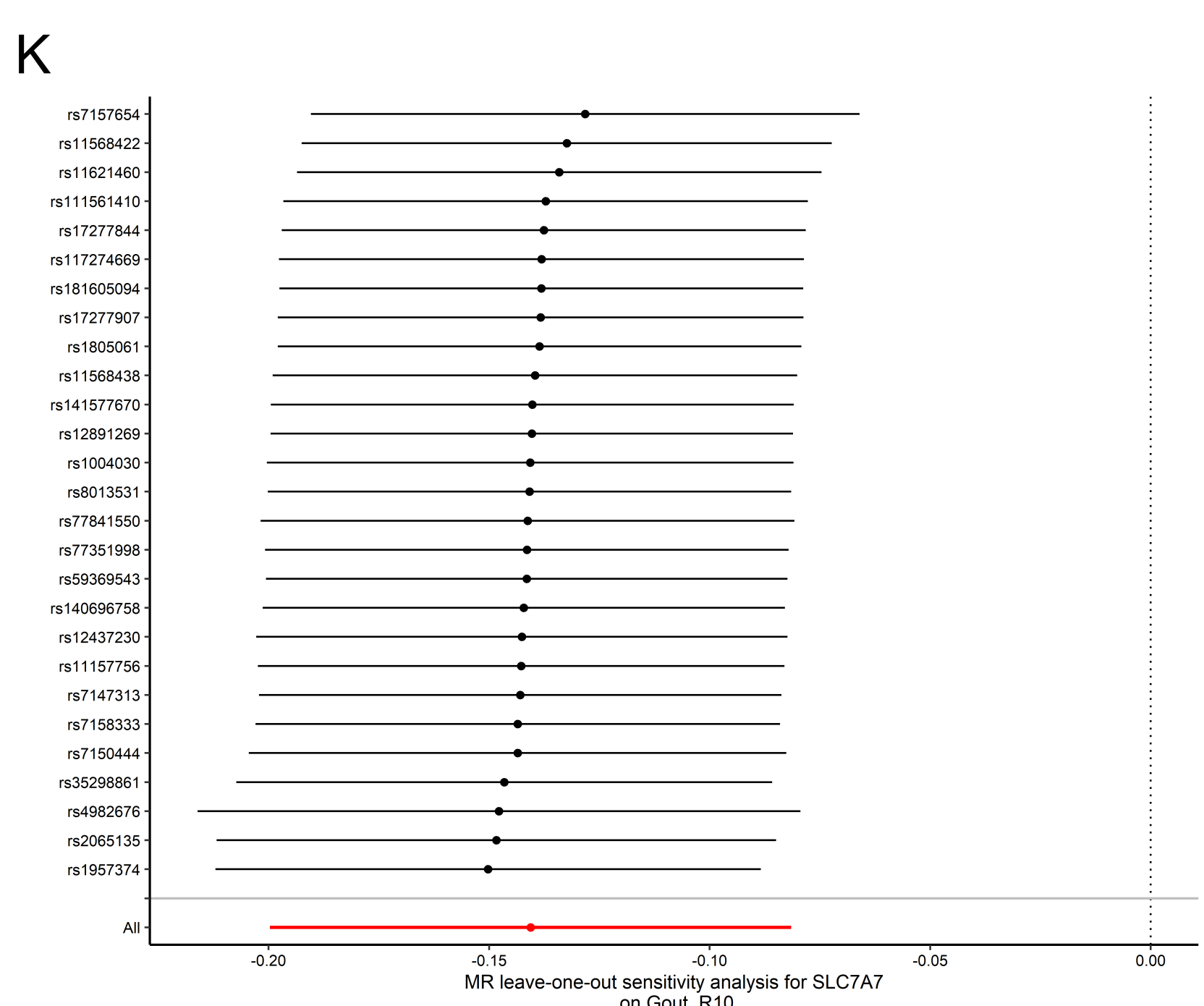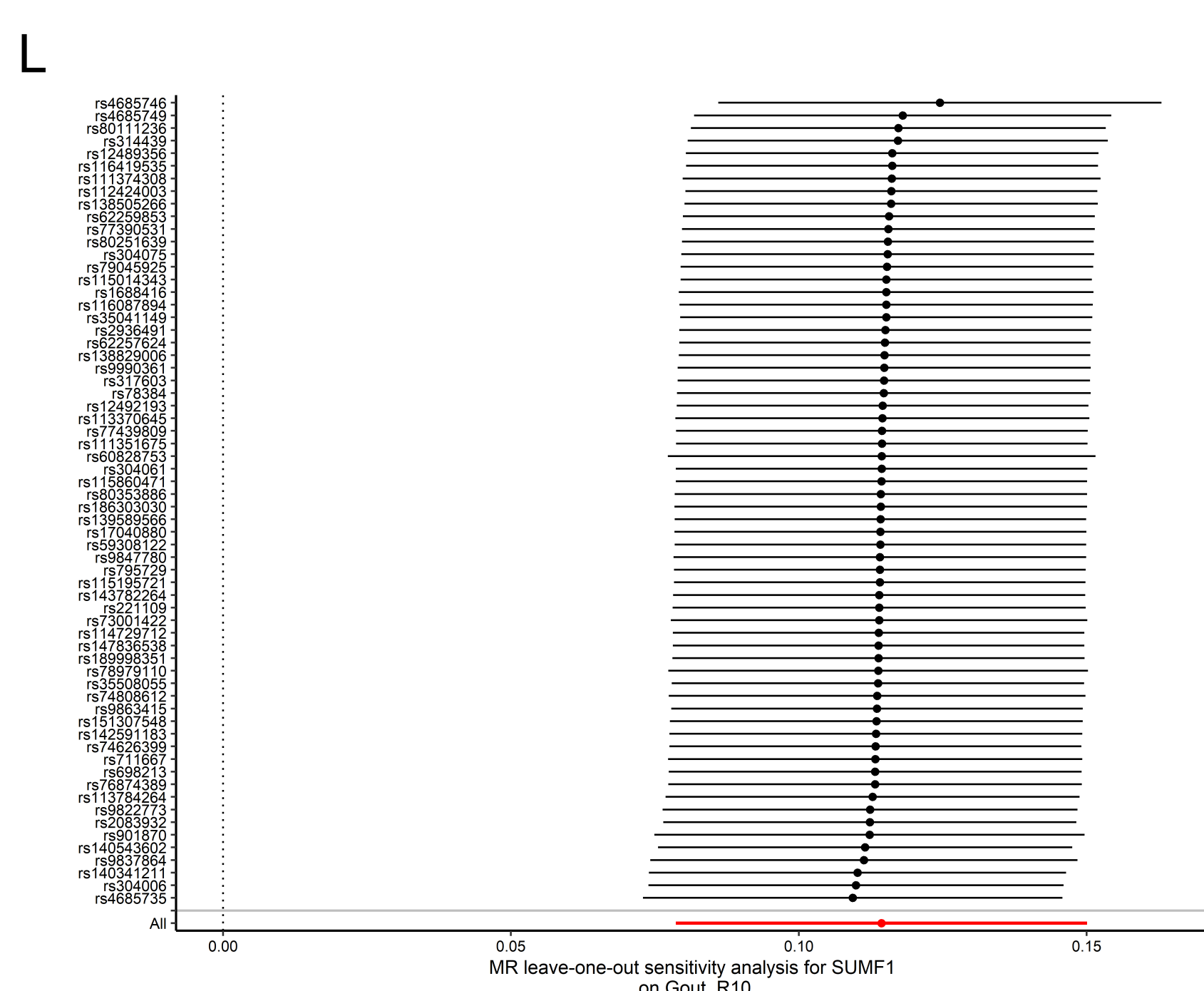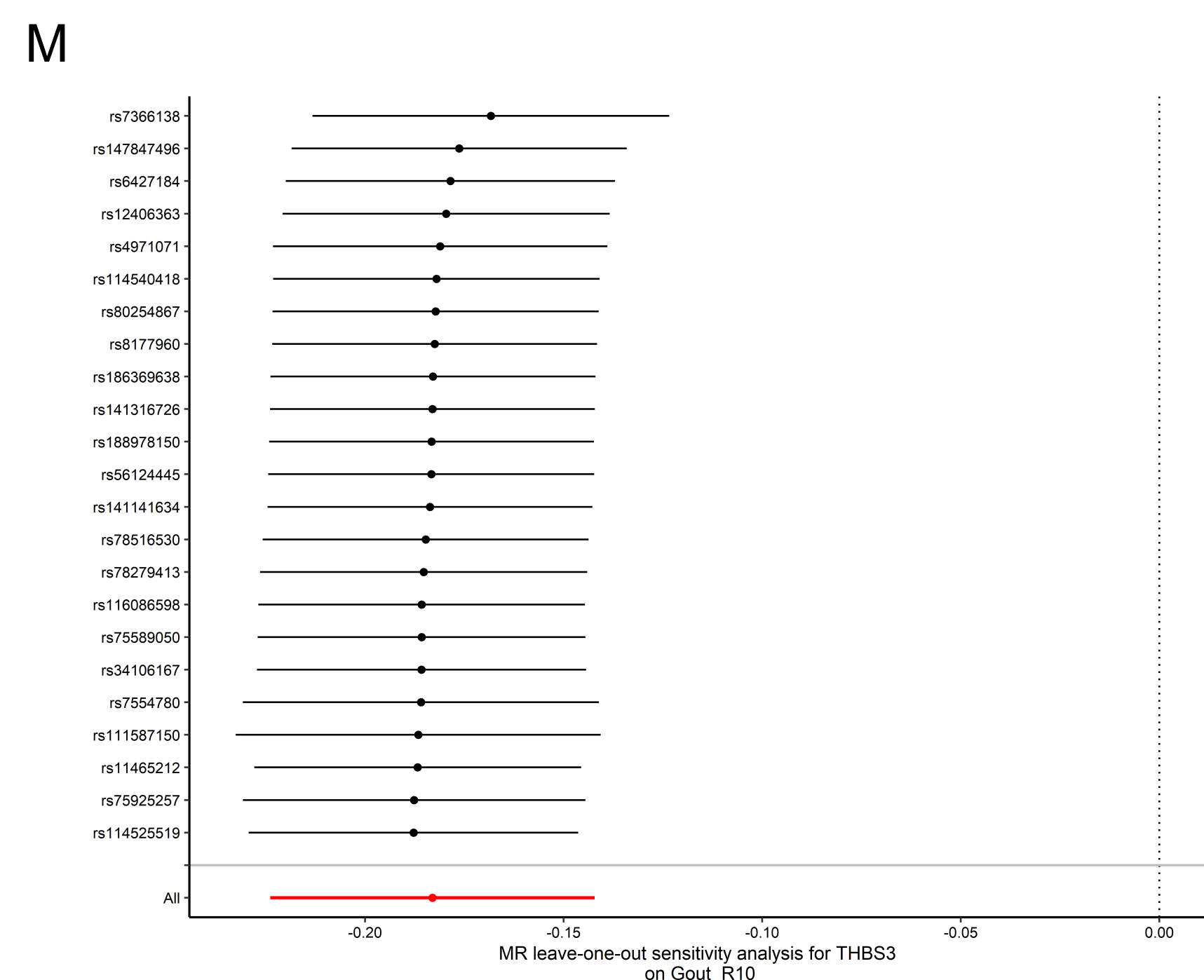

Supplement: Supplementary file 2 — Supplementary Material 2 [file 41065_2024_362_MOESM2_ESM.zip › Figure S2.pdf]

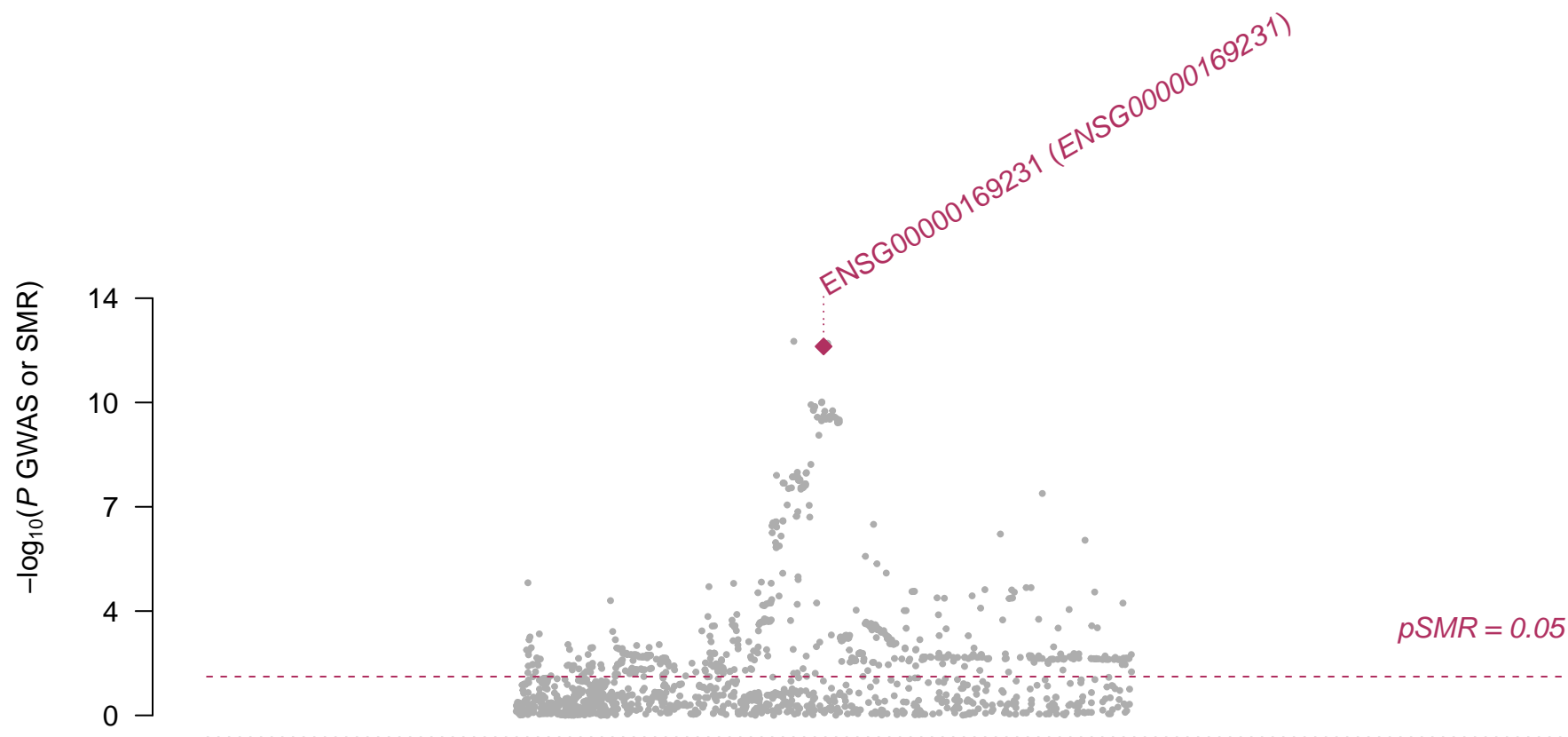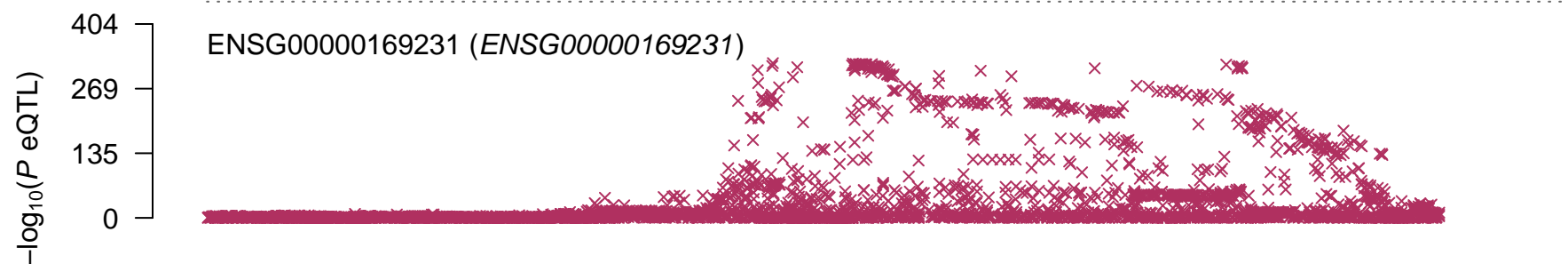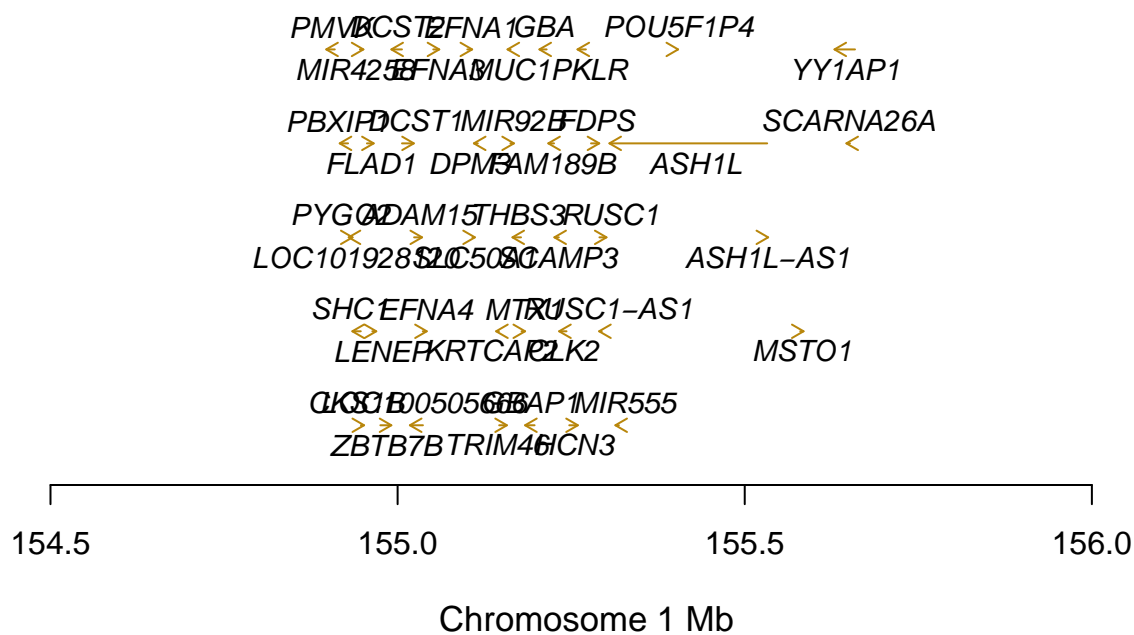

Supplement: Supplementary file 2 — Supplementary Material 2 [file 41065_2024_362_MOESM2_ESM.zip › Figure S3 THBS3.pdf]

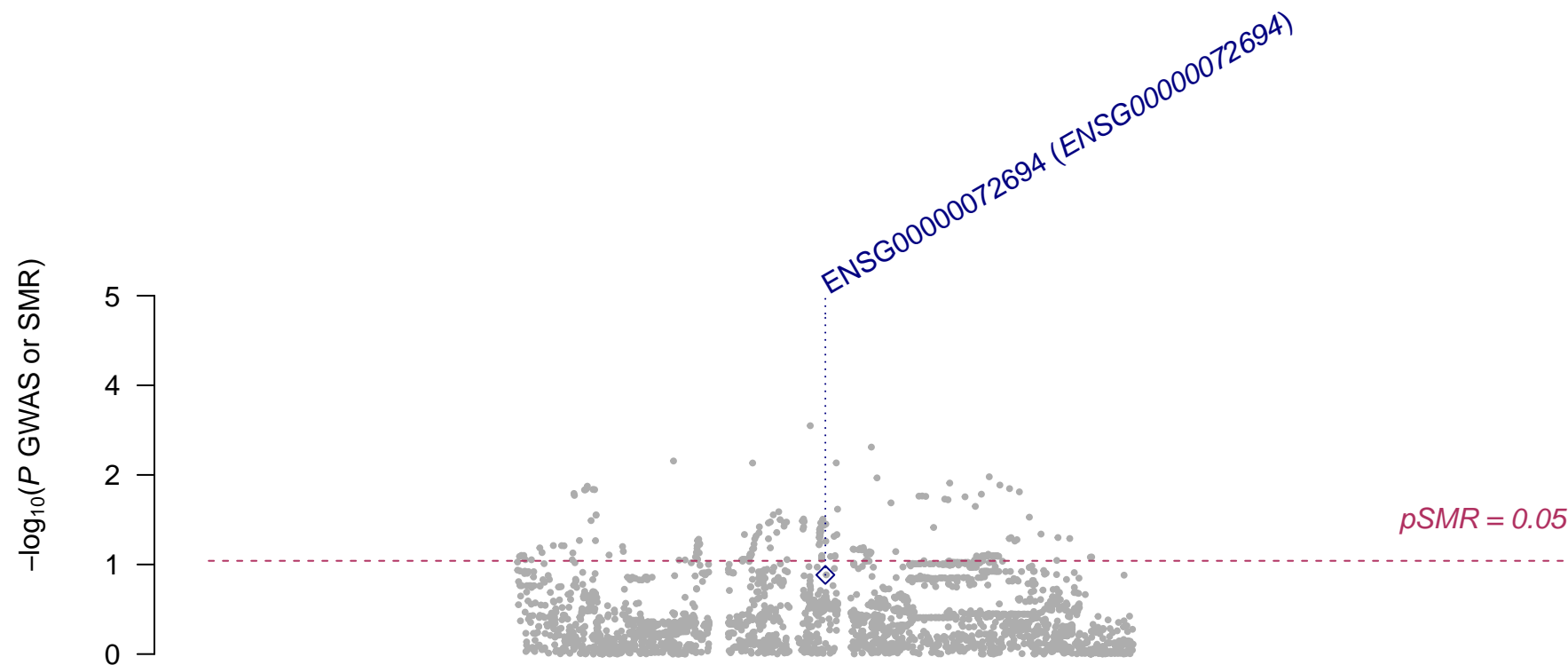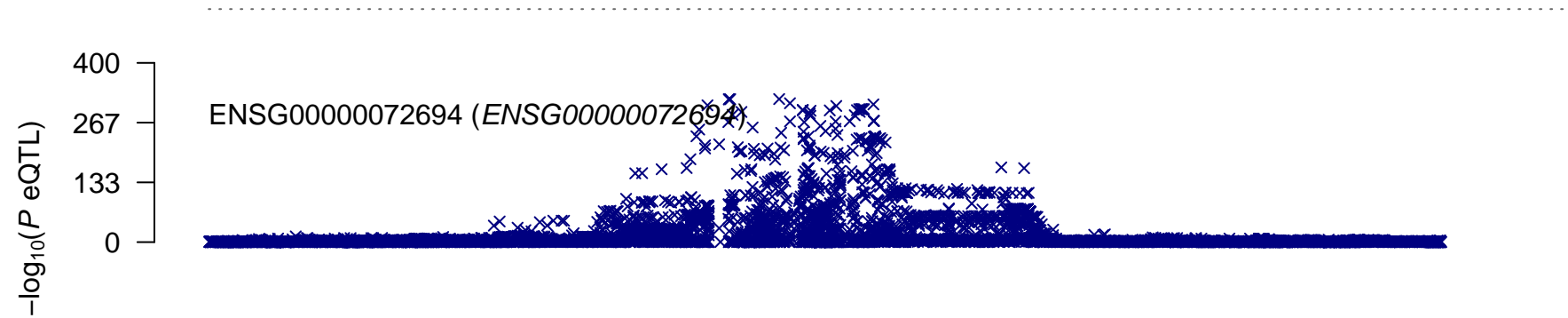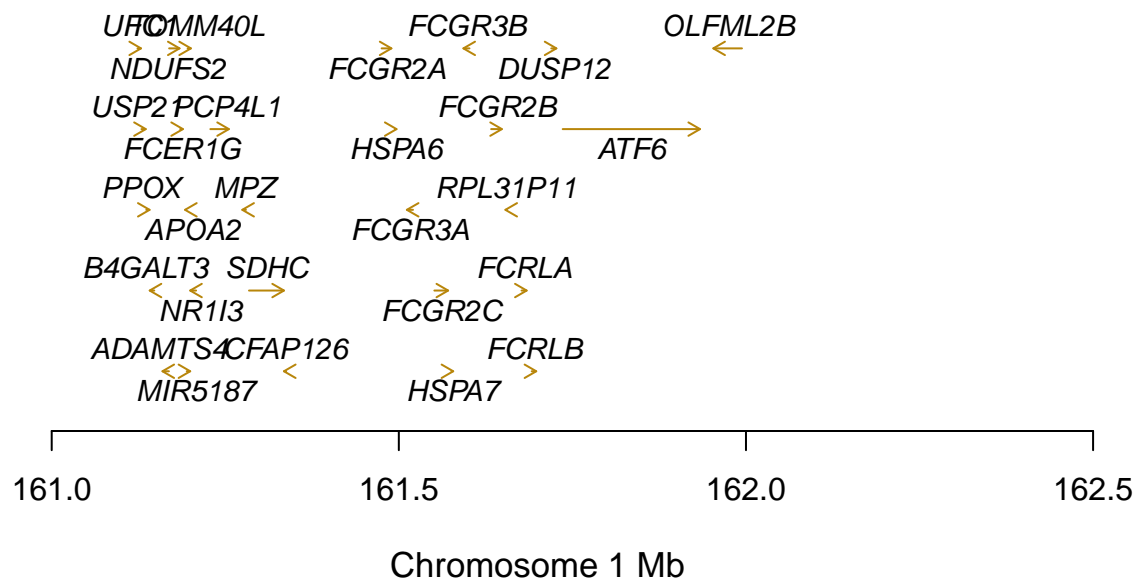

Supplement: Supplementary file 2 — Supplementary Material 2 [file 41065_2024_362_MOESM2_ESM.zip › Figure S4 FCGR2B.pdf]

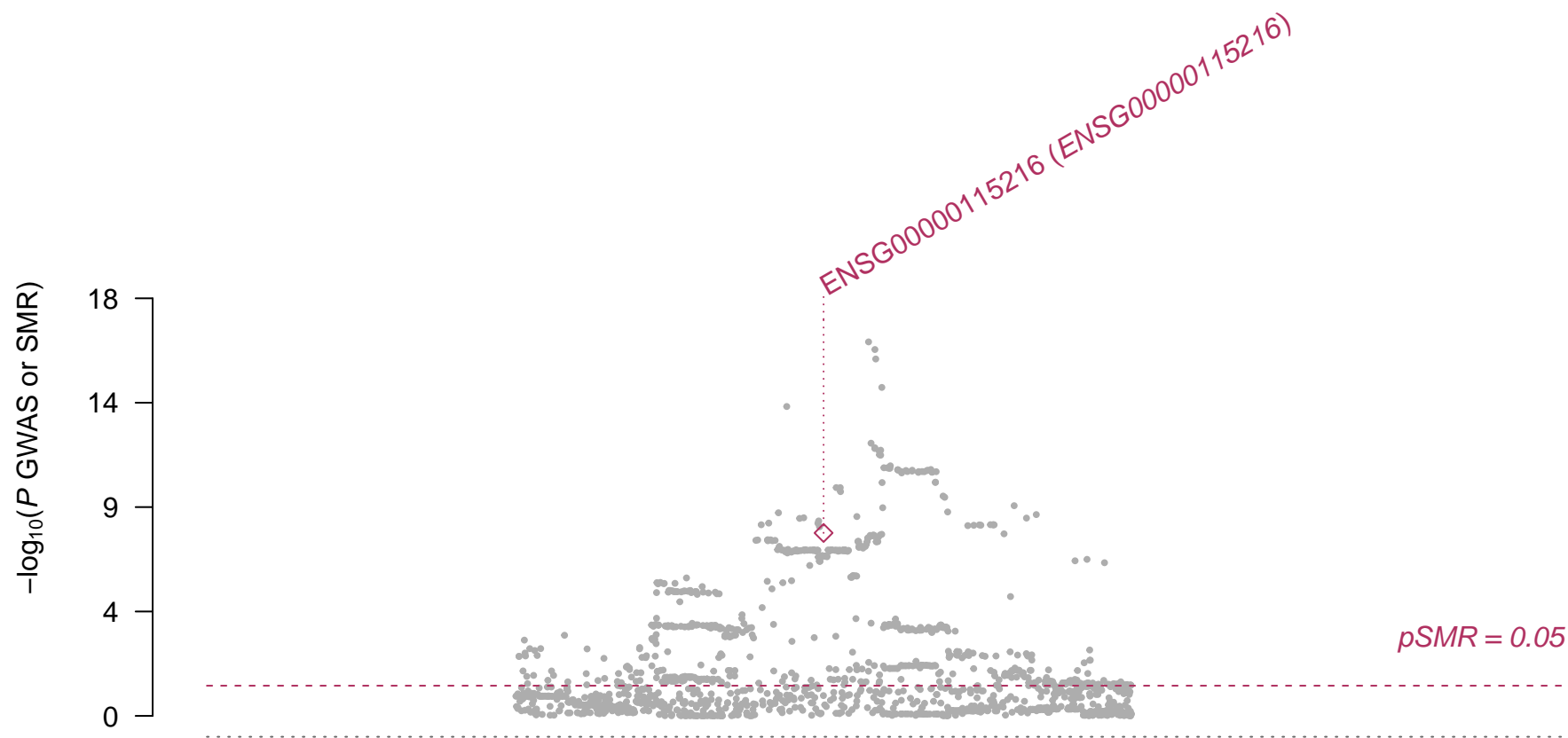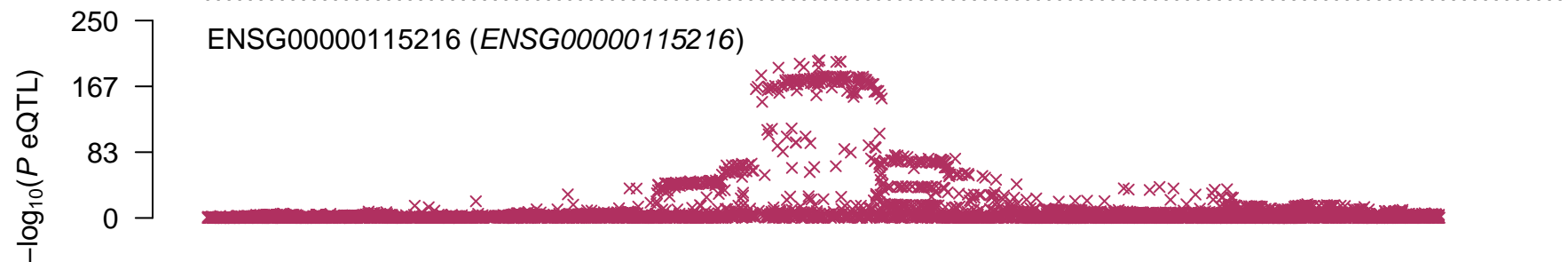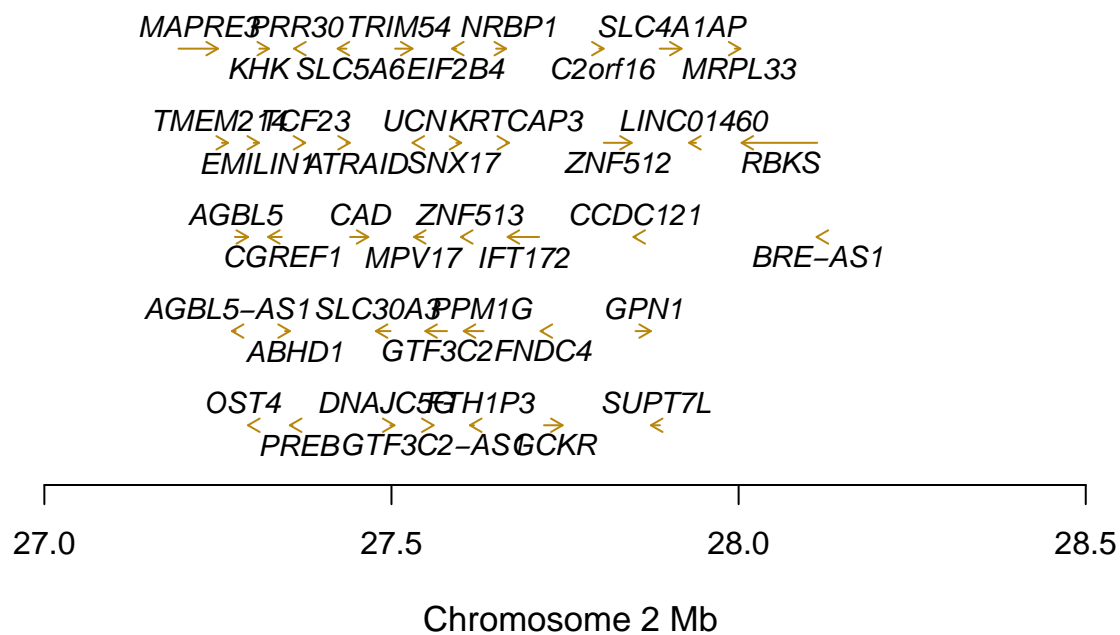

Supplement: Supplementary file 2 — Supplementary Material 2 [file 41065_2024_362_MOESM2_ESM.zip › Figure S6 NRBP1.pdf]

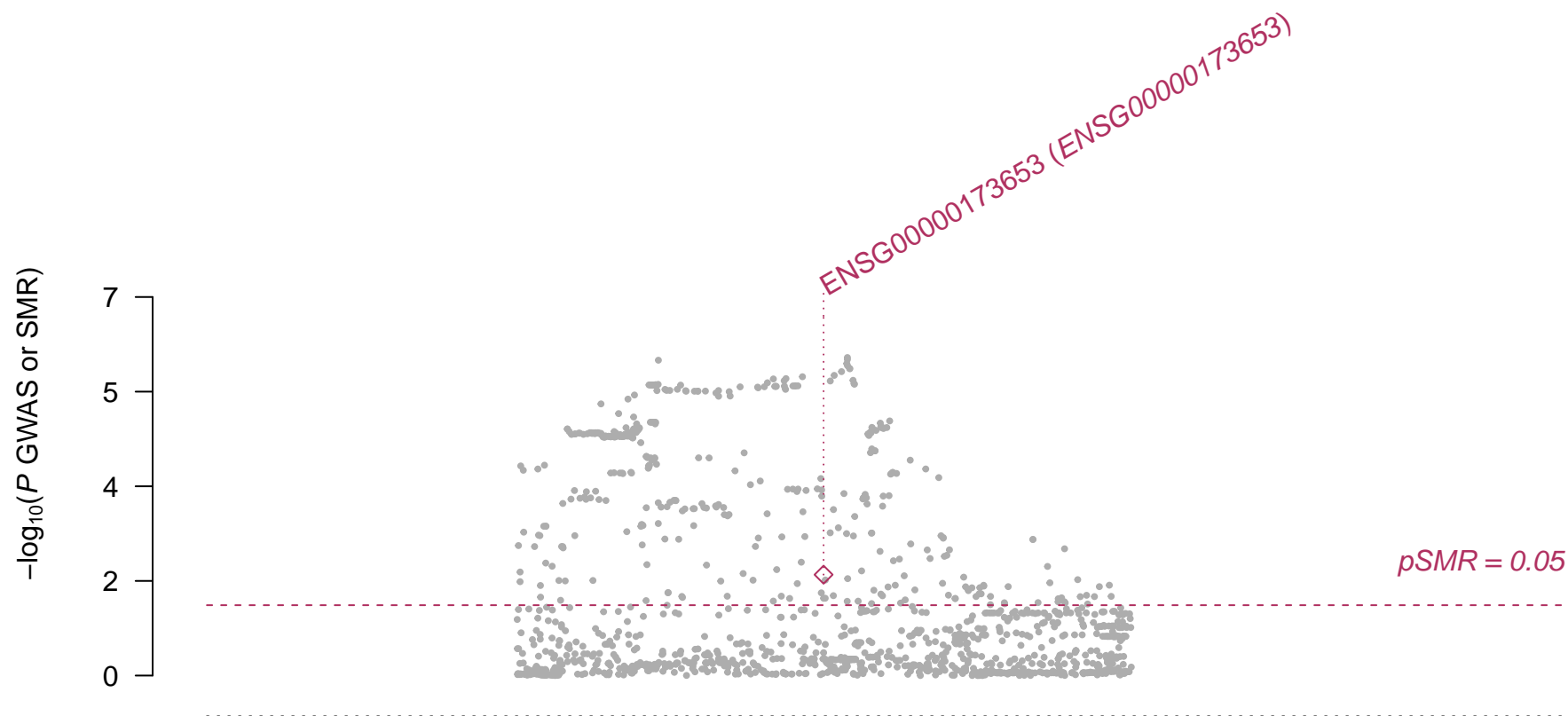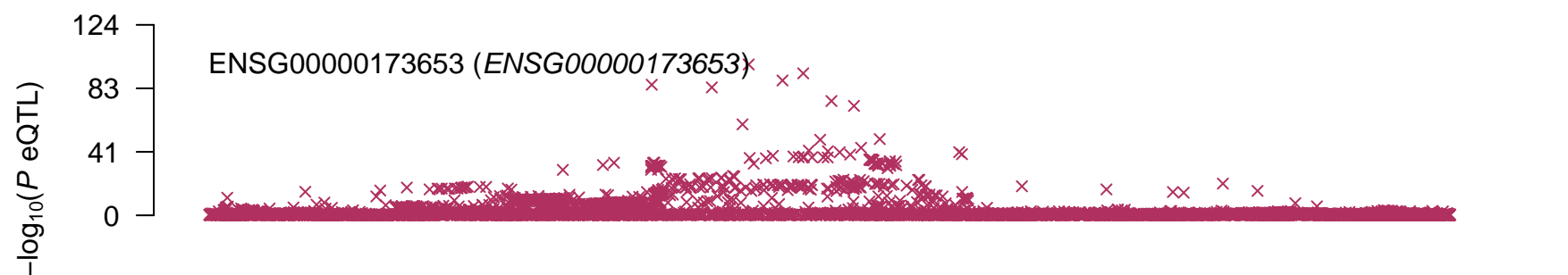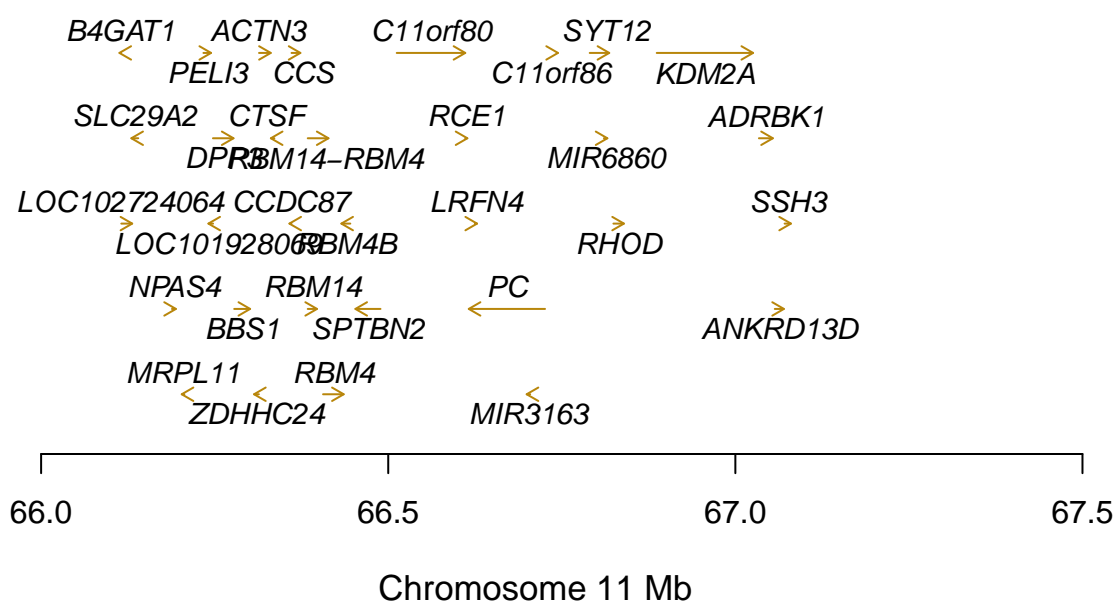

Supplement: Supplementary file 2 — Supplementary Material 2 [file 41065_2024_362_MOESM2_ESM.zip › Figure S7 RCE1.pdf]

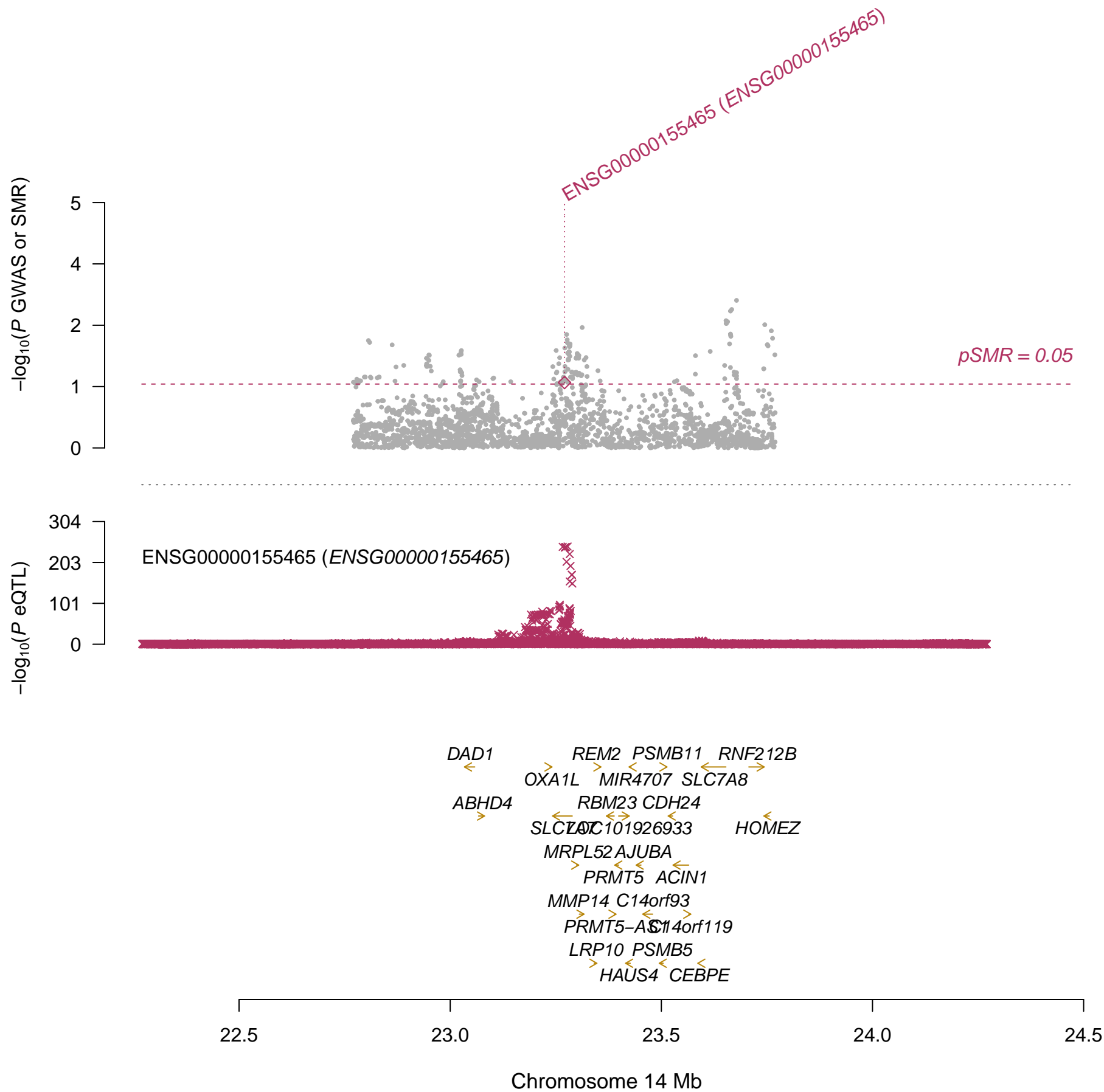

Supplement: Supplementary file 2 — Supplementary Material 2 [file 41065_2024_362_MOESM2_ESM.zip › Figure S8 SLC7A7.pdf]

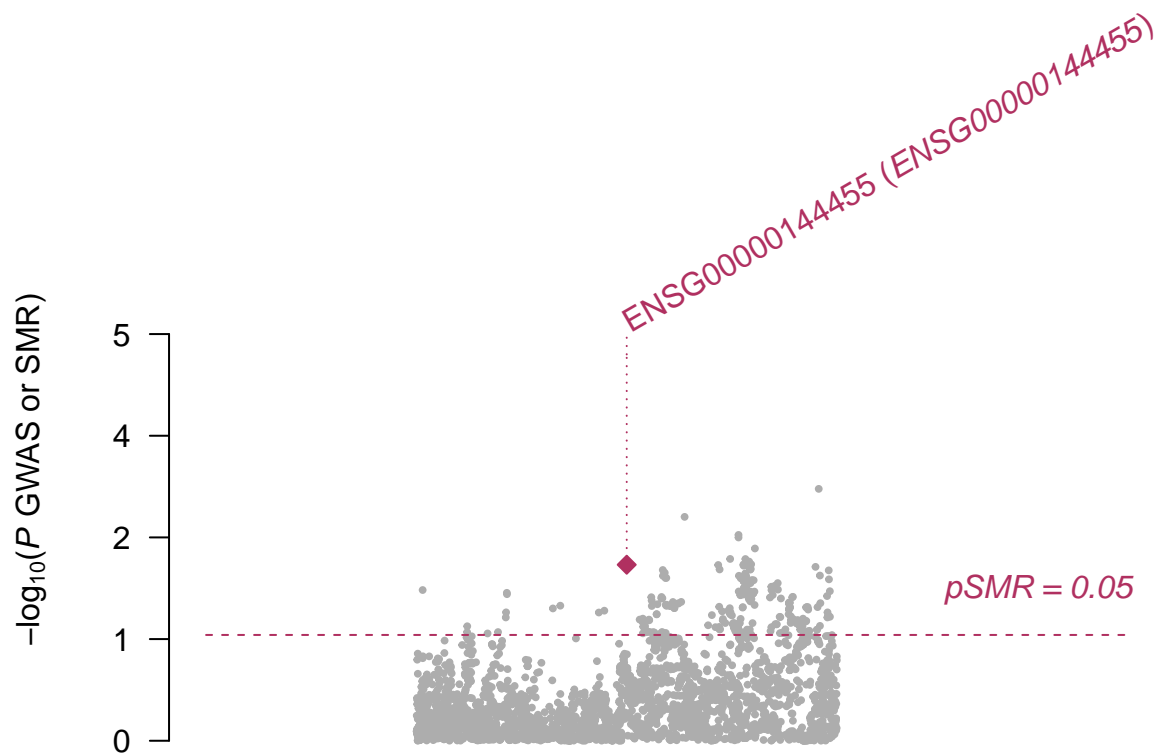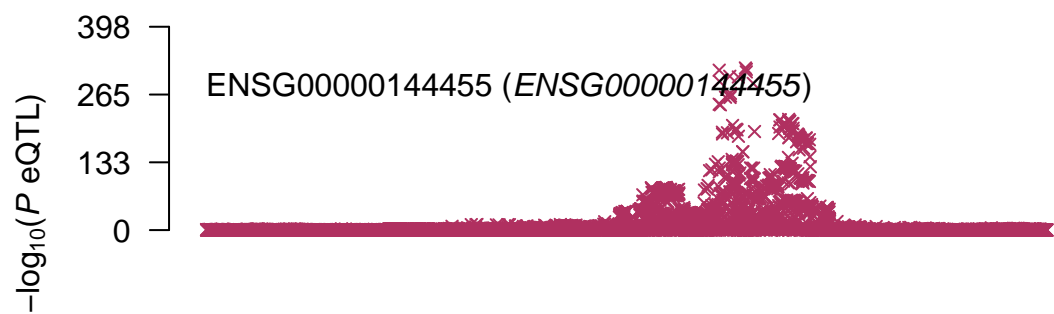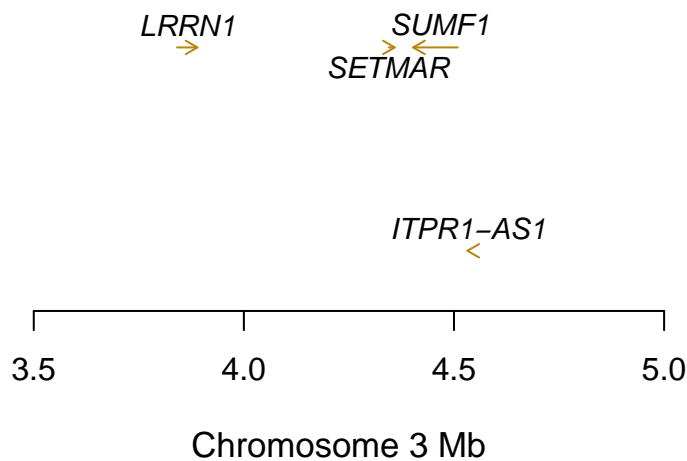

Supplement: Supplementary file 2 — Supplementary Material 2 [file 41065_2024_362_MOESM2_ESM.zip › Figure S9 SUMF1.pdf]
