# Supplementary material for: Genetic insights into therapeutic targets for gout: evidence from a multi-omics mendelian randomization study
Source: Hereditas. 2024 Dec 30;161:56. doi: 10.1186/s41065-024-00362-8 (PMC11684267; doi:10.1186/s41065-024-00362-8)
Supplement: Supplementary file 2 — Supplementary Material 2 [file Figure S5 IL2RB.pdf › Figure S5 IL2RB.pdf]

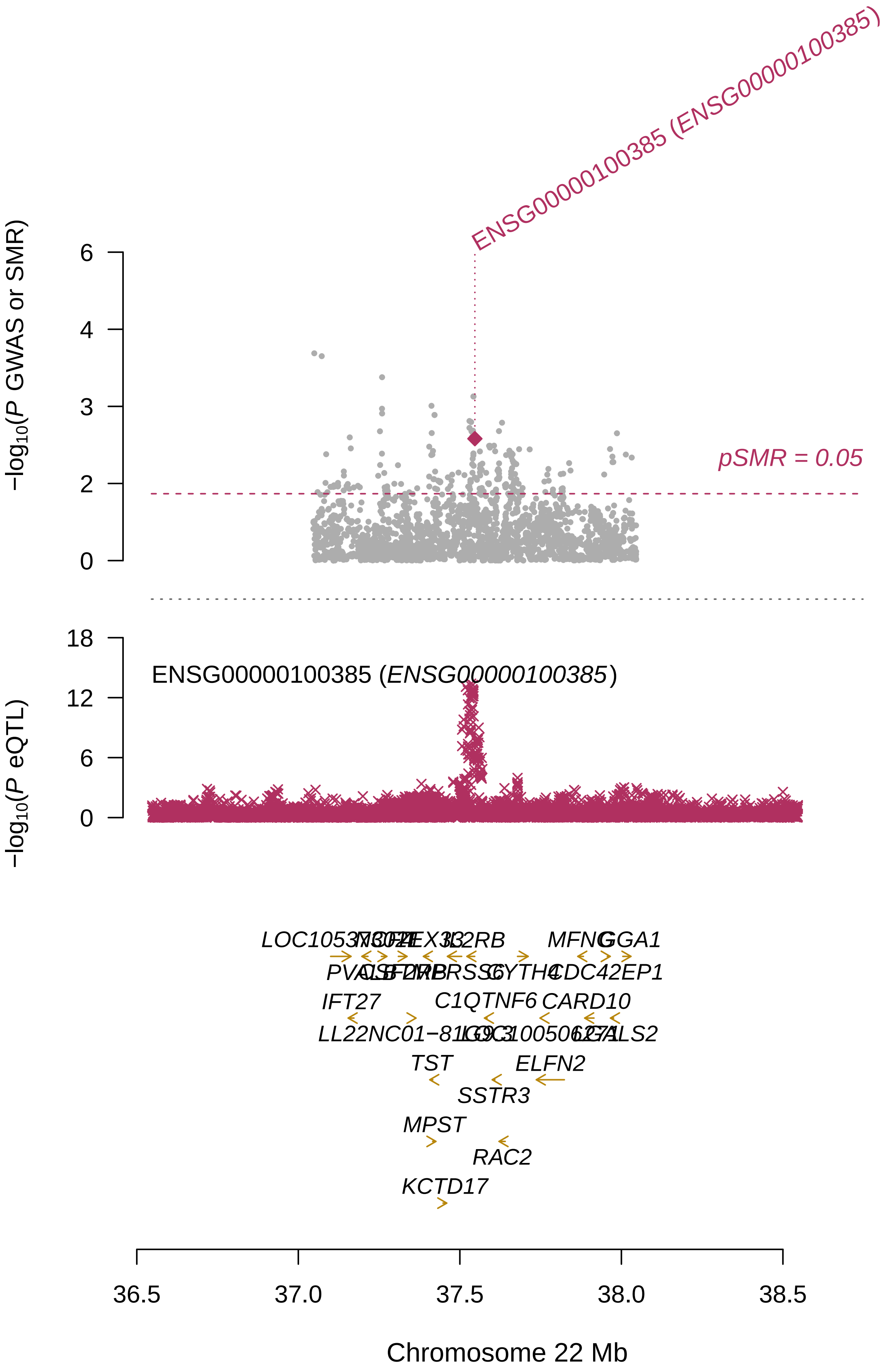

ENSG00000100385 (ENSG00000100385)
pSMR = 0.05
−log10(P GWAS or SMR)
6
4
3
2
0
18
12
6
0
ENSG00000100385 (ENSG00000100385)
−log10(P eQTL)
MFNG
GGA1
CDC42EP1
IFT27
C1QTNF6
CARD10
TST
ELFN2
SSTR3
MPST
RAC2
KCTD17
36.5
37.0
37.5
38.0
38.5
Chromosome 22 Mb
